# Toxoplasma gondii *GRA28* Is Required for Placenta-Specific Induction of the Regulatory Chemokine CCL22 in Human and Mouse

**DOI:** 10.1128/mBio.01591-21

**Published:** 2021-11-16

**Authors:** Elizabeth N. Rudzki, Stephanie E. Ander, Rachel S. Coombs, Hisham S. Alrubaye, Leah F. Cabo, Matthew L. Blank, Nicolás Gutiérrez-Melo, J. P. Dubey, Carolyn B. Coyne, Jon P. Boyle

**Affiliations:** a Department of Biological Sciences, Dietrich School of Arts and Sciences, University of Pittsburgh, Pittsburgh, Pennsylvania, USA; b Department of Pediatrics, University of Pittsburgh School of Medicine, Pittsburgh, Pennsylvania, USA; c Animal Parasitic Diseases Laboratory, Beltsville Agricultural Research Center, Agricultural Research Service, U.S. Department of Agriculture, Beltsville, Maryland, USA; d Department of Molecular Genetics and Microbiology, Duke University School of Medicine, Durham, North Carolina, USA; Harvard T. H. Chan School of Public Health

**Keywords:** CCL22, *Toxoplasma gondii*, placenta, teratogenic, virulence

## Abstract

Toxoplasma gondii is an intracellular protozoan pathogen of humans that can cross the placenta and result in adverse pregnancy outcomes and long-term birth defects. The mechanisms used by T. gondii to cross the placenta are unknown, but complex interactions with the host immune response are likely to play a role in dictating infection outcomes during pregnancy. Prior work showed that T. gondii infection dramatically and specifically increases the secretion of the immunomodulatory chemokine CCL22 in human placental cells during infection. Given the important role of this chemokine during pregnancy, we hypothesized that CCL22 induction was driven by a specific T. gondii-secreted effector. Using a combination of bioinformatics and molecular genetics, we have now identified T. gondii GRA28 as the gene product required for CCL22 induction. GRA28 is secreted into the host cell, where it localizes to the nucleus, and deletion of the GRA28 gene results in reduced CCL22 placental cells as well as a human monocyte cell line. The impact of GRA28 on CCL22 production is also conserved in mouse immune and placental cells both *in vitro* and *in vivo*. Moreover, parasites lacking GRA28 are impaired in their ability to disseminate throughout the animal, suggesting a link between CCL22 induction and the ability of the parasite to cause disease. Overall, these data demonstrate a clear function for GRA28 in altering the immunomodulatory landscape during infection of both placental and peripheral immune cells and show a clear impact of this immunomodulation on infection outcome.

## INTRODUCTION

Toxoplasma gondii is an obligate intracellular parasite that is an important parasite of humans and other animals. While this pathogen is particularly well known to cause severe disease in the immunocompromised, such as those with HIV/AIDS or undergoing immunosuppression for organ transplants, T. gondii is also capable of crossing the placenta and infecting the developing fetus, leading to a variety of infection outcomes, ranging from asymptomatic to severe (1). Importantly, even children born without symptoms are at high risk for extensive health problems later in life, including ocular disease and neurological disorders (2, 3). To date, little is known about how T. gondii gains access to the fetal compartment and how the host responds to the presence of parasites at the maternal-fetal interface.

Recently, we (4) found that primary human trophoblast cells (derived from term placentas) and second trimester placental explants produced the chemokine CCL22 in response to infection with T. gondii (4). Production of this chemokine was dependent on parasite invasion and the dense granule effector trafficking gene product MYR1 (4). While the role of CCL22 during infection with T. gondii is poorly understood, this chemokine is a key molecular signal for the recruitment of regulatory T cells, which are well known for their role in suppressing immune responses to tumors, leading to poor clinical outcomes (5, 6). Importantly, disruption of T_reg_ recruitment to tumors can lead to improved outcomes in animal models. For example, using *Ccl22* DNA vaccines in mice leads to misdirection of regulatory T cells and ultimately reduced tumor growth (5). The role for CCL22 in healthy humans is less well understood, although it is thought to subvert and/or modulate inflammatory responses and may be particularly important for response resolution after pathogen clearance. CCL22 and regulatory T cells also play a critical role during pregnancy, where they seem to govern immune tolerance (7) and regulation of inflammation at the maternal-fetal interface. This regulatory role appears to be critical in determining pregnancy outcome during pathogen-mediated immune activation (7, 8). Given the important role played by CCL22 during pregnancy and our recent findings regarding the ability of a congenitally acquired parasite to directly modulate production of this chemokine, we sought to identify the parasite effector(s) responsible for this in order to determine the impact of CCL22 modulation on congenital transmission and pregnancy outcome during vertical transmission. To do this, we used a bioinformatic screen to identify candidate genes and identified one (*GRA28*; *TGGT1_231960*) as being required for CCL22 induction in human and mouse cells. Overall, these data show that a specific effector is largely responsible for T. gondii-mediated CCL22 induction in a relatively small number of human and mouse cell types and suggest that the manipulation of CCL22 levels by GRA28 may influence the ability of T. gondii to disseminate throughout the host.

## RESULTS

### Toxoplasma gondii induces a monocyte-like cell line to produce the immunomodulatory chemokine CCL22.

**FIG 1:**
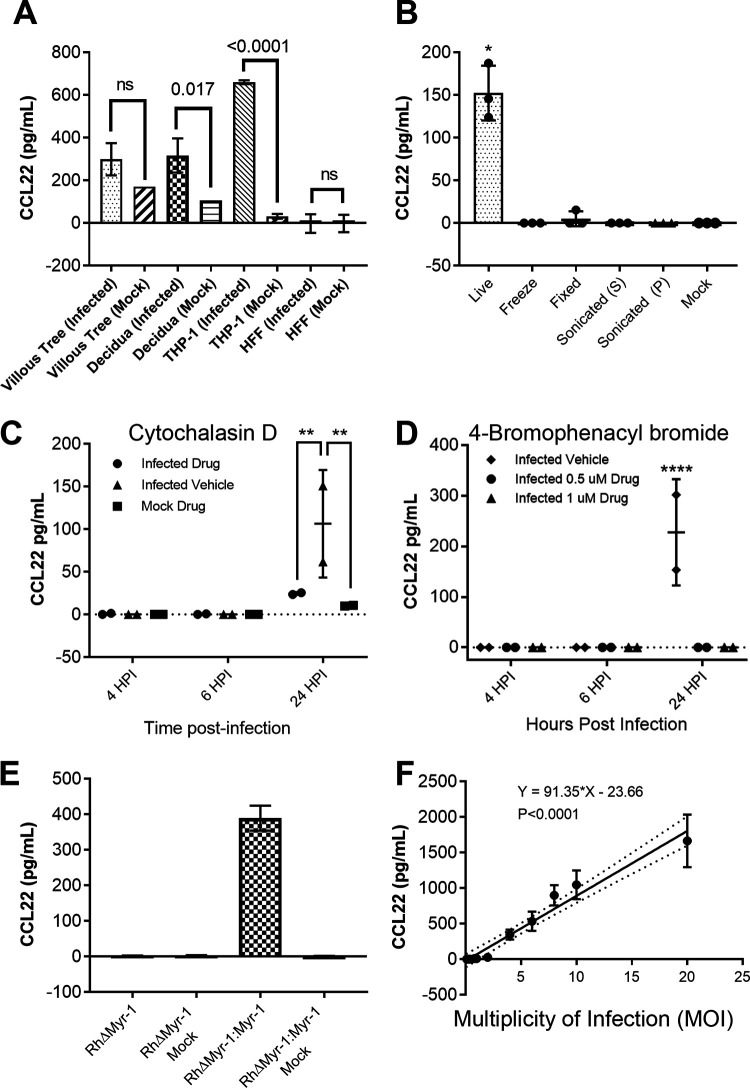
(A) THP-1 cells, human foreskin fibroblasts (HFFs), and second trimester placental samples of villous trees and decidual tissue were infected with a type 1 strain of Toxoplasma gondii (RH:YFP). Statistics on the THP-1 cell and HFF cell samples were performed by ordinary two-way ANOVA with Tukey’s multiple-comparison test. Statistics on the placental samples were performed by two-tailed Welch-corrected *t* tests. (B) Type 1 strain (RH) T. gondii parasites were subjected to multiple treatments as described in Materials and Methods. The soluble fraction of the sonicated treatment is denoted by S; the insoluble fraction is denoted by P. THP-1 cells were then exposed to either live parasites or treated parasites. Statistics were performed by multiple two-tailed Welch-corrected *t* test comparisons with the live parasite treatment at a *P *of *≤*0.0145. (C and D) Type 1 strain (RH) T. gondii parasites were treated with either cytochalasin D (C) or 4-bromophenacyl bromide (D) as described in Materials and Methods. THP-1 cells were then infected with the respective parasite treatment. Statistics were performed by two-way ANOVA and multiple-comparison *post hoc* tests, where the Cyt-D *P *value is 0.009, and the 4-BPB *P* value is <0.0001. (E) Type 1 strain (RH) T. gondii parasites deficient in Myr-1 (TgRHΔ*Myr-1*) and their complement (TgRHΔ*Myr-1*:*Myr-1*_c_) were used to infect THP-1 cells. (F) Type 1 strain (RH) T. gondii parasites were used to infect THP-1 cells at MOIs of 20, 10, 8, 6, 4, 3, 1, 0.8, 0.4, 0.2, and 0.1. (A to D) The respective cells/tissues were infected at a multiplicity of infection (MOI) of 3. Supernatants were collected at 24 h postinfection unless indicated otherwise and assayed by CCL22 ELISA.

Previous work established that placental explants and primary human trophoblasts infected with T. gondii had increased *CCL22* transcript abundance and released more CCL22 protein into the culture medium than mock-infected controls (4). Since we also found that not all cell types produce CCL22 in response to infection (e.g., human foreskin fibroblasts [HFFs]), we were interested in identifying a human cell line that could be used as a more tractable model than placental cells to assay T. gondii-driven CCL22 induction. THP-1 cells, a cell line derived from a patient with monocytic leukemia, were a reasonable candidate given their origins in the myeloid lineage and known production of CCL22 in response to a variety of stimuli (9, 10). We infected THP-1 cells with a type 1 T. gondii strain (RH88 or RH*YFP* [4]) at a multiplicity of infection (MOI) of 3. Following 24 h of infection, supernatants were collected from each well. HFFs were infected in parallel as negative controls. Mock treatments involved passing the parasite solution through a 0.22-μm-pore-size filter prior to exposure to the cells. Based on a CCL22 ELISA, T. gondii infection induced CCL22 in THP-1 cells, and as expected there was no CCL22 production from mock-treated controls or T. gondii-infected HFFs (Fig. 1A). We also infected primary placental tissues in the same manner, and as expected, villous tree explants and decidua taken from second trimester placentas produced significantly more CCL22 than mock-treated controls (Fig. 1A). In addition to the type 1 RH strain, other T. gondii strain types (type 2, strain PRU, and type 3, strain CEP) (see Fig. S1A in the supplemental material) also induced secretion of CCL22 from THP-1 cells, as did the nearest extant relative of T. gondii, Hammondia hammondi (Fig. S1B). In contrast to *H. hammondi*, and just as we observed previously in primary human placental cells (4), Neospora caninum had no effect on THP-1 production of CCL22 (Fig. S1B). These data provided strong support that the mechanism of CCL22 induction is the same for THP-1 and placental cells.

10.1128/mBio.01591-21.2 FIG S1 (A) Type 1 (RH88), type 2 (PRU), and type 3 (CTG) T. gondii strains all induce CCL22 in THP-1 cells. For mock treatment, cells were treated with 0.2-μm-pore-size-filtered parasite suspensions. (B) *H. hammondi* induces CCL22 production in THP-1 cells while N. caninum does not in comparison to mock-treated cells. (C) The T. gondii gene *MYR1* is required for CCL22 production by THP-1 cells after infection. T. gondii Δ*MYR1* parasites were compared to Δ*MYR1*:*MYR1*-complemented parasites, and only the *MYR1*-complemented parasites induced secretion of CCL22 from the wild-type (black data points; left) or *MYD88* knockout (blue data points; right) THP-1 cells. While *MYD88* knockout cells produced significantly less CCL22 in response to *MYR1*-complemented parasites, they still produced CCL22 at levels much greater than Δ*MYR1*-infected cells or mock-treated cells. *MYR1* knockout and complemented parasites were kindly provided by John Boothroyd and Michael Panas, Stanford University. Download FIG S1, JPG file, 0.5 MB. Copyright © 2021 Rudzki et al. 2021 Rudzki et al. https://creativecommons.org/licenses/by/4.0/ This content is distributed under the terms of the Creative Commons Attribution 4.0 International license.

We also determined if live parasites were required to induce CCL22 in THP-1 cells by exposing host cells to parasites that were exposed to a variety of lethal treatments. As shown in Fig. 1B, dead parasites failed to induce CCL22 production by THP-1 cells. We also pretreated parasites and host cells with 10 μg/ml cytochalasin D (Cyt-D) to block invasion (11), and as shown in Fig. 1C, Cyt-D-treated parasites were significantly impaired in their ability to induce CCL22, suggesting that active invasion was required for this phenomenon. We obtained similar results with the inhibitor 4-bromophenacyl bromide (4-BPB) (Fig. 1D), which also significantly blocked CCL22 production by THP-1 cells at 0.5 and 1 μM. This drug blocks rhoptry and dense granule secretion from T. gondii but not microneme secretion (12), suggesting that the factor is not a microneme protein (Fig. 1D). We also infected THP-1 cells with T. gondii parasites that were deficient in the dense granule trafficking protein MYR1 (13) (kind gift from John Boothroyd, Stanford University) and compared them to T. gondii RHΔ*MYR1*:*MYR1_c_* (TgRHΔ*MYR1*:*MYR1_c_*) parasites. TgRHΔ*MYR1* parasites failed to induce any detectable CCL22 from THP-1 cells, while, as expected, TgRHΔ*MYR1*:*MYR1_c_* parasites induced significantly more than mock-treated cells (Fig. 1E). We also observed a very tight correlation between parasite multiplicity of infection (MOI) and CCL22 levels, suggesting that the signal was driven primarily by the parasite rather than the host cell (Fig. 1F). Based on these results, we felt confident that the unknown secreted factor driving CCL22 production in human primary placental cells was very likely the same as the one driving it in the THP-1 cell line and chose THP-1 cells for screening candidate effectors given their tractability in the laboratory.

### Transcript abundance correlation analysis identifies a large group of putatively MYR1-trafficked gene products.

**FIG 2:**
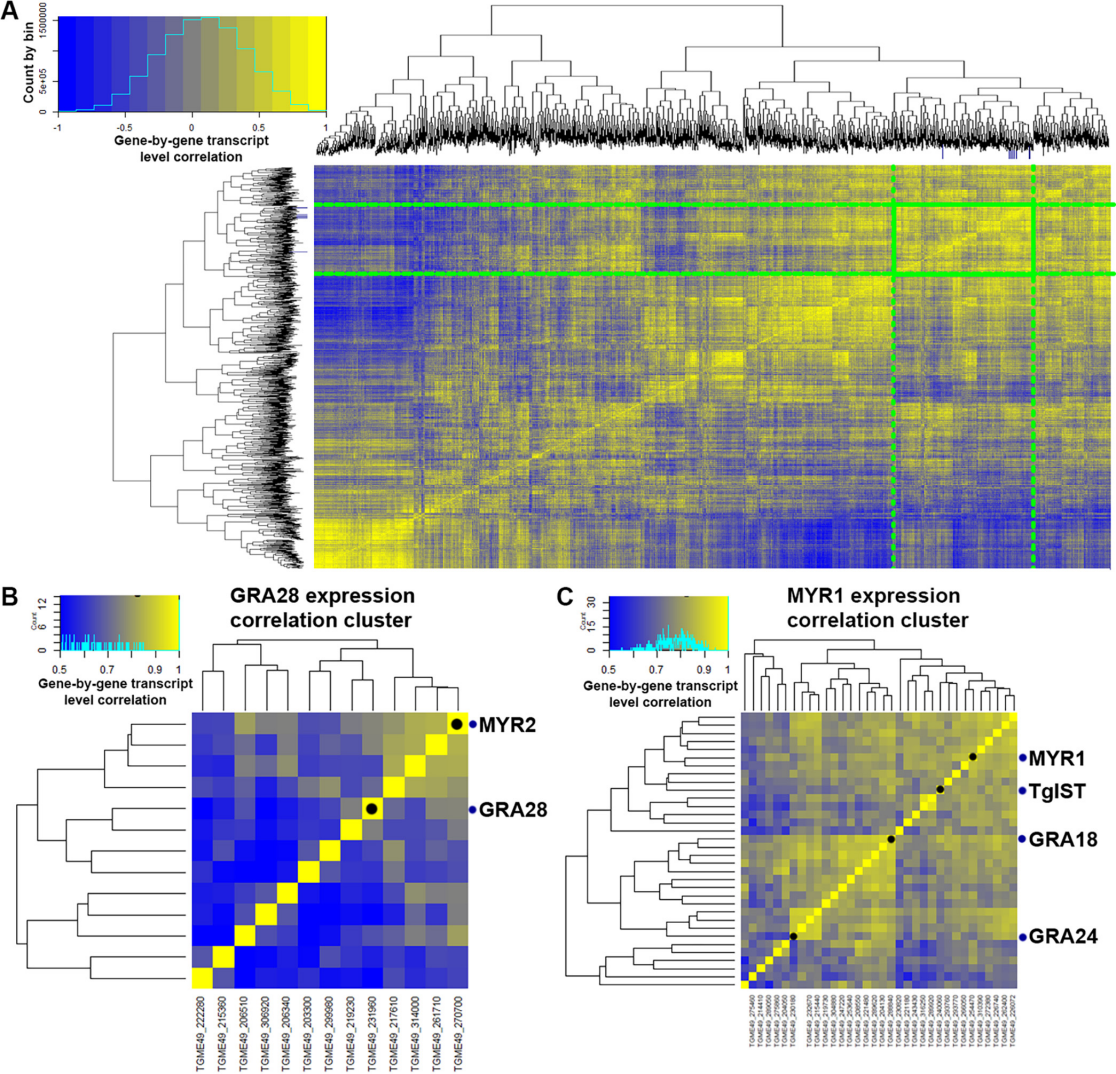
Transcript correlation analyses to identify putative MYR1 substrates based on 396 publicly available microarray data sets. (A) Gene-by-gene correlation data for T. gondii genes across 396 microarray data sets. A subset (3,217 genes with at least one sample having a normalized log_2_-transformed value of ≥10) of the total number (8,058) of T. gondii genes is shown for simplicity. Genes outlined in the green box indicate the cluster containing all of the bait genes as well as candidate CCL22-inducing genes, with the exception of *Toxofilin*. Dark blue tick marks on each dendrogram indicate the location of all of the bait genes. The color scale covers correlations ranging from −1 to +1. (B) Subcluster containing *MYR2* and *GRA28*. (C) Subcluster containing *MYR1*, *TgIST*, *GRA18*, and *GRA24*. It is important to note that for panels B and C, the color scale is from 0.5 to 1.0 to highlight subcluster differences.

As described previously (above and in reference 4), we have determined that primary human trophoblast cells infected with T. gondii have a transcriptional signature that is characterized by the production of immunomodulatory chemokines, with CCL22 being the most potently induced. To identify candidate T. gondii genes responsible for this effect on placental cells, and since this effect required the T. gondii effector translocation complex protein MYR1 (4), we hypothesized that MYR1-dependent substrates would have highly correlated gene expression profiles across diverse gene expression data sets. To test this hypothesis, we generated an “all versus all” correlation matrix of 396 T. gondii Affymetrix microarray data sets that we downloaded and curated from the Gene Expression Omnibus (see Materials and Methods). Analysis of the entire correlation matrix (shown in Fig. 2A and downloadable at https://doi.org/10.6084/m9.figshare.16451832) confirms this hypothesis for certain gene classes. For example, we identified one cluster containing multiple SAG-related sequences (SRS) which are typically expressed at high levels in bradyzoites (including SRS49 cluster members A, C, and D) (Fig. S2A) and another containing 70 genes, 43 of which encode ribosomal subunits (Fig. S2A). Examination of the gene expression heat maps across all 396 microarray analyses clearly show distinct patterns of gene expression in these two clusters depending on life stage treatment exposure (Fig. S2A and B).

10.1128/mBio.01591-21.3 FIG S2 Clusters of coregulated genes share developmental expression profiles and functional activities. (A) Transcript abundance correlation analysis (left) and clustered transcript abundance analysis (in RMA log_2_-normalized units) for 21 genes with transcripts known to increase in abundance during the tachyzoite-to-bradyzoite transition, including *BAG1*, *LDH2*, and the enolase gene. The bar across the top of the expression heat map indicates the life cycle stage source for each of the samples. The pie chart indicates that 19 of the 21 transcripts increase in abundance during pH-induced bradyzoite development according to the bradyzoite differentiation (3-day time series) data set at https://toxodb.org. (B) Cluster containing multiple ribosomal protein coding genes (for both the large and small subunits) showing high transcript abundance in tachyzoites and bradyzoites but comparatively low transcript abundance in samples taken from sporozoites and merozoites. Download FIG S2, JPG file, 1.7 MB. Copyright © 2021 Rudzki et al. 2021 Rudzki et al. https://creativecommons.org/licenses/by/4.0/ This content is distributed under the terms of the Creative Commons Attribution 4.0 International license.

We quantified the degree of transcript abundance correlation between 5 “bait” genes (*MYR1*, *MYR2*, and *MYR3* [13, 14] and the known *MYR1*-dependent substrates TgGRA24 and TgIST [15, 16]) and all other genes across all 396 expression data sets. We identified genes as candidate MYR1 substrates if they had an average correlation with the 5 “bait” genes of ≥0.7, a *dN*/*dS* ratio of ≥2, and the presence of a predicted signal peptide or at least one transmembrane domain. Using this set of filters, we were left with 28 candidate genes (plus all 5 bait genes, which also met these cutoffs), including the known TgMYR-dependent substrate TgGRA25 (17). Since all known MYR1 trafficked substrates are dense granule proteins, we eliminated any surface antigens or soluble enzymes, leaving a number of confirmed dense granule proteins (e.g., GRA4 and GRA8) and conserved hypothetical proteins. Importantly, when we examined the correlation between the bait genes and all T. gondii genes annotated as “dense granule” either in the primary product notes or via user annotation, we found that not all dense granule-encoding transcripts correlated highly with bait transcript levels (Fig. S3A), indicating that our approach could discriminate between different classes of proteins secreted from the same organelle. For example, while genes like *GRA32* (*TGME49_212300*) had transcript levels with relatively high (>0.8) correlations with bait transcript levels, other genes encoding GRA1, GRA2, and GRA11 paralogs had transcript levels that correlated much more poorly with the bait genes. This is despite the fact that most of these dense granule-encoding genes have high transcript levels compared to other gene clusters, as shown in the heat map in Fig. S3A, demonstrating that our approach yielded an additional layer of discrimination to categorize dense granule-trafficked gene products. Moreover, many of the coregulated genes are not yet annotated, but based on our analysis, one would predict that many are likely to be dense granule protein-derived secreted effectors or structural constituents of this parasite organelle.

10.1128/mBio.01591-21.4 FIG S3 Transcript level correlation analysis with the 6 bait genes used in this study (A) and clustered, normalized gene expression data (B) for all genes in the T. gondii genome annotated with the term “dense granule” in the product name or user comments. Dense granule protein coding genes fall into two major clusters in the correlation analysis, with the top cluster containing the known secreted effectors GRA15, GRA24, TgIST, GRA7, and GRA28. Download FIG S3, JPG file, 1.1 MB. Copyright © 2021 Rudzki et al. 2021 Rudzki et al. https://creativecommons.org/licenses/by/4.0/ This content is distributed under the terms of the Creative Commons Attribution 4.0 International license.

### T. gondii *GRA28* is the gene responsible for CCL22 induction in human immune and placental cells.

**FIG 3:**
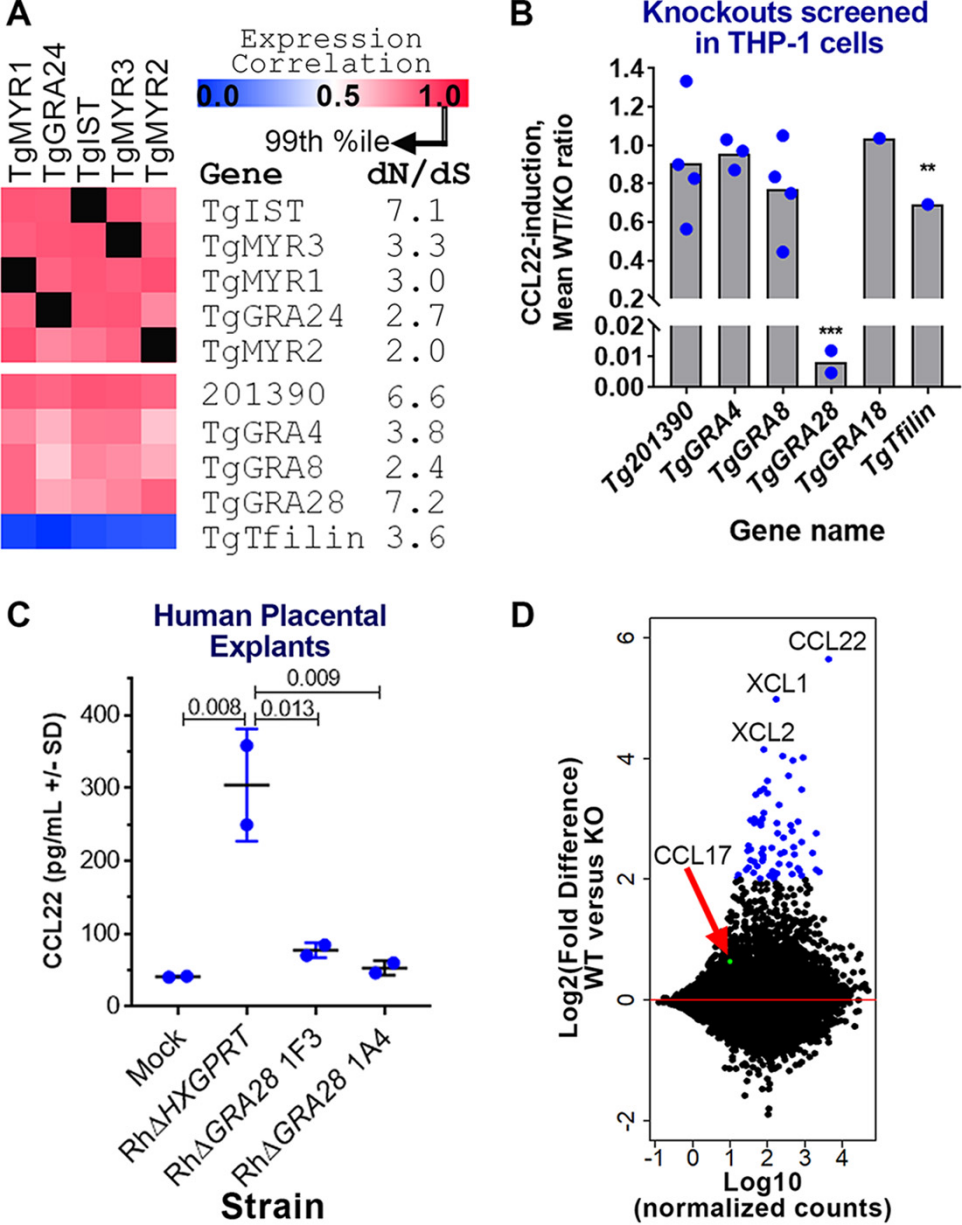
Identification of the T. gondii gene *GRA28* as an inducer of CCL22 in human cells. (A) Gene expression correlations across 396 T. gondii expression microarrays between Tg*MYR1* and 4 additional “bait” genes (top) and 5 candidate CCL22-inducing effectors (bottom). *dN*/*dS* ratios are also shown to illustrate the high level of positive selection acting on this class of genes. (B) Effect of deleting 5 candidate genes on CCL22 secretion in THP-1 cells, showing that Δ*GRA28* parasites induced significantly less CCL22 than wild-type controls (>100-fold reduction; ***, *P* < 0.0001). PRUΔ*Toxofilin* parasites also induced significantly lower levels of CCL22 in THP-1 cells (1.4-fold reduction; **, *P* < 0.01). Each blue dot indicates a genetically distinct knockout clone. (C) Δ*GRA28* parasite clones also induce significantly less CCL22 from primary human second trimester placental villous explants. (D) MA plot (where the log_2_ fold change is on the *y* axis and the log-transformed average of normalized counts is on the *x* axis) of RNA-seq analysis performed on THP-1 cells infected with WT or Δ*GRA28* T. gondii (RH strain). CCL22 and the chemokines CXL1 and CXL2 were the most highly GRA28-dependent transcripts, while a handful (*n* = 64) of other genes had significantly higher transcript abundance in WT parasites than in Δ*GRA28* parasites (*P*_adj_ < 0.001; log_2_FC > 2; blue symbols). CCL17 (red arrow, green symbol), a chemokine that is typically coregulated with CCL22, did not show any evidence of being induced by GRA28.

When we specifically examined correlations between the bait genes listed above and *MYR1*, we found that *MYR1* expression profiles were highly correlated at the transcriptional level with *MYR2/3* and *IST* (Fig. 2B and C and Fig. 3A, top), consistent with the idea that MYR1 substrates could be identified using this approach. After identifying a small list of candidate genes (Fig. 3A, bottom), we deleted each using CRISPR-CAS9, validated them using diagnostic PCR and in some cases by sequencing (Fig. S4), and screened them for CCL22 induction in THP-1 monocytes by ELISA. Among the five genes that we tested (including *GRA18*, which was recently found to induce Ccl22 in mouse macrophages [18]), we found that *GRA28* (*TGME49_231960*) was required for the induction of CCL22 secretion by infected THP-1 monocytes (Fig. 3B). We also found that Δ*Toxofilin* parasites had significantly reduced levels of CCL22 induction (Fig. 3B), albeit to a much lesser extent than Δ*GRA28* parasites. We think it likely that this decrease is owed to the reduced invasion capacity of Δ*Toxofilin* parasites rather than a direct impact of this gene product on host CCL22 production (19, 20).

10.1128/mBio.01591-21.5 FIG S4 Validation of knockouts generated for the present study (A to D) and for the *GRA18* gene, which was generated in another study (E) (H. He, M.-P. Brenier-Pinchart, L. Braun, A. Kraut, *et al.*, eLife 7:e39887, 2018, https://doi.org/10.7554/eLife.39887). Clones that were validated as knockouts and used in the CCL22 induction assays shown in Fig. 3B are labeled with boldface italics. We validated them using two parallel approaches for most of the knockouts: (i) amplification across the site targeted by the protospacer(s) encoded by the transfected gRNA-expressing plasmid(s), where no amplification indicated a potential insertion of plasmid sequence into that location, and (ii) amplification across the entire coding sequence of the gene, where no amplification also suggests insertion of the selectable marker and another plasmid sequence at at least one of the protospacer sites. In some cases, multiple protospacer-encoding plasmids were transfected into the same parasite population (protospacer numbers are our own internal nomenclature), and in these cases, it was possible to have insertion/disruption at both protospacer sites (and this occurred in some instances). Primer sequences and gRNA target sites are shown in Table S1. (A) Validation of four TGGT1_201390 knockout clones generated by batch transfection with gRNAs targeting two distinct sites in the TGGT1_201390 coding sequence (2 and 12). (Left gel) Two primer sets (A and B) were used to amplify across the site targeted by the protospacer, and MAF1b primers were used as a positive control. Clones D11 and G11 likely had insertions at protospacer site 2 (as evidenced by the lack of amplification with primer set A), while clone D2 likely had an insertion in protospacer site 12, as evidenced by a lack of amplification with primer set B. Clone F2 had amplification of the correct size at both protospacer sites (2 and 12), but we could not amplify the coding sequence from the F2 clone (right gel, lane labeled F2), suggesting that the locus was disrupted in this strain as well. All four of these knockout clones were assayed in biological triplicate for CCL22 induction in THP-1 cells (Fig. 3B). (B) Validation of three GRA4 knockout clones out of five tested. Clones A1:D6 and B2:C1 (where A1 and B1 indicate that the parasites were from distinct transfections) had a likely insertion in the *GRA4* gene at protospacer site 0, while clone B2:B11 had a likely insertion at both sites (0 and 26). (C) Validation of four *GRA8* knockout clones. Parasites were transfected with a single gRNA-expressing plasmid (targeting gRNA sequence 4), and PCR of all 4 clones failed to amplify across the gRNA 4-target site. All amplifications across the gRNA target site 27 worked, as did the positive control amplification of MAF1b. (D) Validation of four *GRA28* knockout clones. Parasites were cotransfected with plasmids carrying gRNAs targeting sites 5 and 26 and queried using primers targeting the entire locus (sets A and B in this case) or flanking gRNA target sites 5 and 26 (sets C and D). PCR across the entire *GRA28* locus for clones 1A4, 1D4, and 1D3 all failed to generate PCR products, suggesting that these three clones had disruptions in the *GRA28* gene. PCR of clone F3 with primer sets A and B gave a product of the expected size. PCR across the gRNA target site 26 failed for clone 1A4, while PCR across gRNA target site 5 failed for clones 1D4 and 1D3. Taken together, these data indicate that clones 1A4, 1D4, and 1D3 were all *GRA28* knockouts via insertion of selectable marker and accompanying plasmid sequences at the targeted gRNA sites. For clone 1F3, the locus seemed to be intact, but when we sequenced PCR products similar to those amplified by primer sets C and D (at gRNA target sits 5 and 26, respectively) we determined that clone 1F3 had a single-base-pair deletion at base 250 relative to the start codon of *GRA28* which introduced a stop codon 100 bp downstream of the indel (as well as multiple stop codons in frame further downstream). Sequences across the gRNA target site 26 were identical to the wild type. The deletion near gRNA target site 5 was within the gRNA protospacer sequence itself, just proximal to the PAM site (GTTCCGCTGGTGCCTT**C**ACC [TGG] was mutated to GTTCCGCTGGTGCCTT_ACC [TGG]). Therefore, we treated 1F3 as a *GRA28*-null parasite strain. (E) Validation of *GRA18* knockouts received from the Bougdour lab. We received *GRA18* knockout and wild-type clones from the laboratory of Alexandre Bougdour and validated them using PCR. In this case, we generated primers to amplify across the entire *GRA18* locus, which was completely deleted using double homologous recombination with large sequences flanking the entire coding region. As expected, we could amplify across the entire *GRA18* locus in PRU WT and *GRA18*-complemented strains but failed to do so in the *GRA18* knockout. Download FIG S4, PDF file, 0.5 MB. Copyright © 2021 Rudzki et al. 2021 Rudzki et al. https://creativecommons.org/licenses/by/4.0/ This content is distributed under the terms of the Creative Commons Attribution 4.0 International license.

10.1128/mBio.01591-21.1 TABLE S1 List of primers and protospacer sequences used to generate and validate knockouts for the present study. (Column 1) Targeted locus indicated either by a ToxoDB gene model number or the name of the annotated gene if available. (Column 2) Locus primers were chosen to span the entire coding sequence of the targeted gene and used in PCRs to determine if there was an insertion of the pSAG:gRNA plasmid and/or the selectable marker (in this case, the *HXGPRT* gene) somewhere in the coding region. PCRs with locus primers would fail based on the fact that the size of the predicted product would be incompatible with the extension times used to amplify the wild-type locus (see the legend for Fig. S4 and Fig. S4 itself for more details). (Column 3) gRNA(s) (protospacer-adjacent motif [PAM]). The protospacer sequence encoded within the gRNA along with the PAM presents in the target location in the genome. All protospacers used in the present study are shown for each targeted locus. (Column 4) As outlined in the legend for Fig. S4, we also used PCR across each site targeted by the gRNA(s) as a secondary means to determine if the locus was disrupted. As for the locus primers, PCRs would fail if the pSAG:gRNA plasmid and/or the selectable marker had inserted into the site targeted by the gRNA. Download Table S1, DOCX file, 0.01 MB. Copyright © 2021 Rudzki et al. 2021 Rudzki et al. https://creativecommons.org/licenses/by/4.0/ This content is distributed under the terms of the Creative Commons Attribution 4.0 International license.

To determine if GRA28 was responsible for CCL22 production by human placental cells, we infected second trimester human villous placental explants with wild-type (WT) and Δ*GRA28* parasites and observed a marked decrease in CCL22 production by explants exposed to Δ*GRA28* parasites compared to those exposed to the WT (Fig. 3C). To gain a broader understanding of the transcriptional networks altered by *GRA28*, we compared THP-1 cells infected with RHΔ*HPT*:*HPT* and RHΔ*GRA28* using transcriptome sequencing (RNA-seq). A relatively small number of transcripts had significantly altered abundance when stringent statistical cutoffs were used {67 genes with an adjusted *P* value (*P*_adj_) of <0.0001 and an absolute log_2_ fold change [abs(log_2_FC)] of ≥2 are highlighted in Fig. 3D}, and these included CCL22 as well as the chemokines XCL1 and XCL2. Interestingly, transcript abundance for CCL17, a chemokine that is often coregulated with CCL22 (21, 22) and which is induced in some cells along with CCL22 by the T. gondii effector GRA18 (18), was not dependent on *GRA28* (Fig. 3D). The majority of transcripts that were GRA28 dependent were of higher abundance in the WT than in the Δ*GRA28* parasites when slightly relaxed statistical cutoffs were used (263 higher, 33 lower; *P*_adj_ of <0.05 and log_2_FC of ≥1 or ≤1).

**FIG 4:**
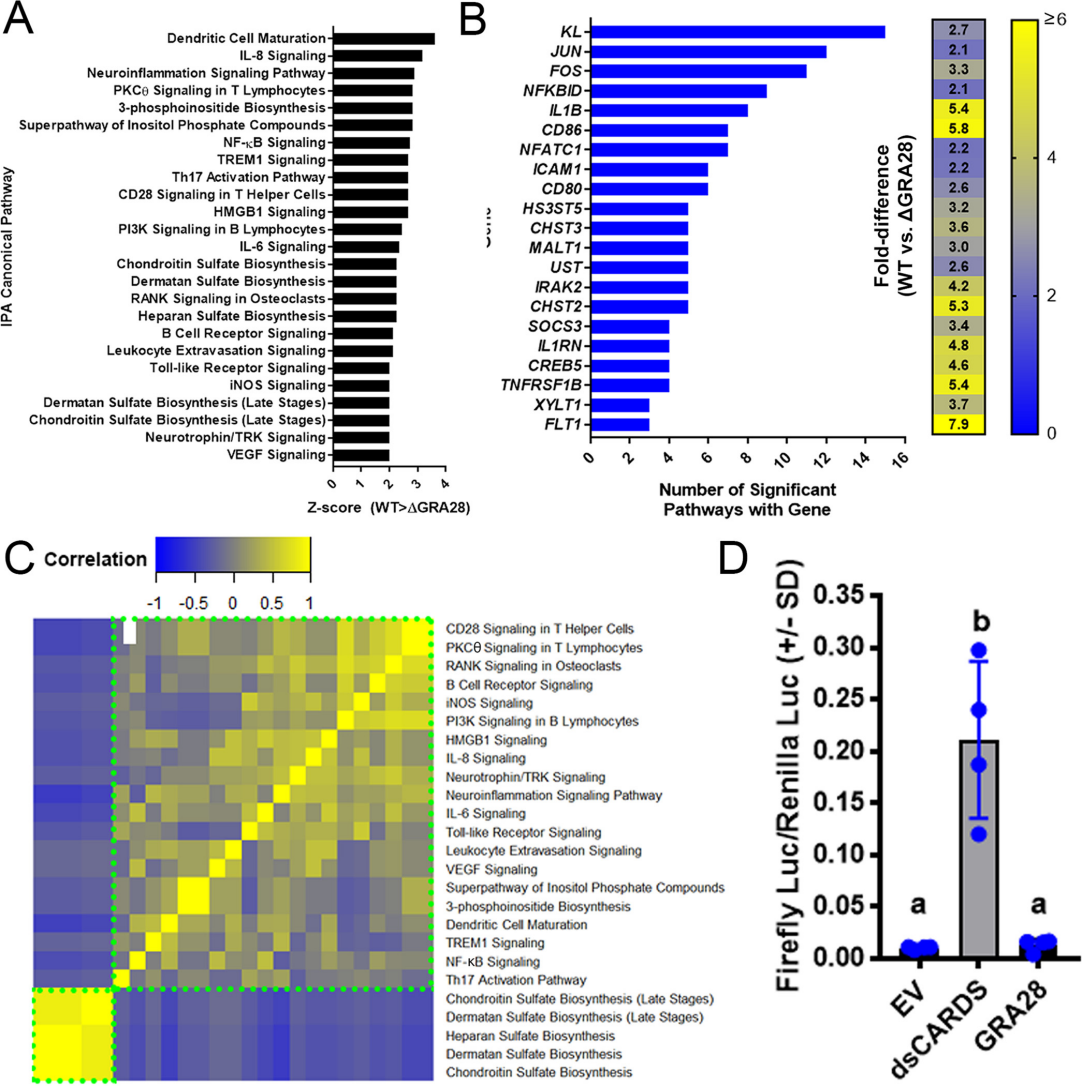
IPA analysis of THP-1 cells infected with WT or Δ*GRA28* T. gondii parasites for 24 h showing canonical pathways that were differentially regulated (−log(*P*) ≥ 2; Z-score, less than or equal to −2 or greater than or equal to 2) depending on the presence or absence of the GRA28 gene. (A) Z-scores for significant canonical pathways. All were higher in WT than in Δ*GRA28* T. gondii. (B) There was extensive overlap of component genes within each canonical pathway, particularly for the genes *KL* and those encoding components of the AP-1 transcription factor complex (*JUN* and *FOS*). A heat map of fold difference in transcript abundance for cells infected with RH WT and RHΔ*GRA28* is shown. (C) GRA28 is responsible for driving transcriptional changes in two major gene clusters identified based on the degree of gene sharing between each canonical pathway (clusters outlined in dotted green boxes). The larger cluster consists primarily of immunity-related pathways, while the smaller cluster consists of genes involved in proteoglycan synthesis. (D) Quantification of NF-κB activation in 293T cells. Cells were transfected with NF-κB firefly luciferase plasmid, a constitutive *Renilla* luciferase plasmid, as well as empty vector (EV), a construct expressing a CARD domain (dsCARDS), or the first exon of T. gondii GRA28. While the CARD domain construct induced firefly luciferase expression as expected, expression of T. gondii GRA28 had no significant impact on firefly luciferase levels (letters indicate groups that were not significantly different from one another according to one-way ANOVA and Tukey’s multiple-comparison *post hoc* test.).

We performed pathway analysis on these sets of regulated genes using Ingenuity pathway analysis (IPA) and identified host cell pathways that were either more or less induced in WT T. gondii-infected cells compared to RHΔ*GRA28*-infected cells (Fig. 4), including dendritic cell maturation, interleukin 6 (IL-6) and IL-8 signaling, and NF-κB signaling (Fig. 4A). When we assessed the degree of gene overlap in these gene sets, we found that 10 of the pathways contained the *JUN* and *FOS* genes (Fig. 4B), indicating a potential role for AP-1 complex-targeted transcripts in GRA28-dependent transcriptional changes. We examined correlations across these gene sets (after creating a matrix of presence/absence of each of the genes shown in Fig. 4B) and identified two nonoverlapping sets of genes. The larger cluster contains multiple immunity-related genes, while the smaller cluster contains genes involved in proteoglycan synthesis (Fig. 4C), including the *XYLT1* gene, which encodes the enzyme that adds UDP-xylose to serine residues as a first step in glycosaminoglycan synthesis. When we performed a similar analysis using the “upstream regulator” module in IPA, we identified a small set of significant (Z-score ≥ 2; *P < *0.001) regulatory factors that were upstream of the GRA28-dependent gene set, including multiple regulators associated with the NF-κB pathway (Fig. S5A). Cluster (Fig. S5B) and downstream gene overlap (Fig. S5C) analyses further confirmed the *FOS* and *JUN* genes as contributing to the signaling pathways that were GRA28 dependent, while also confirming a putative role for NF-κB. For example, the cluster with the most similar target gene overlap contains multiple genes in the NF-κB pathway (*NFKBIA*, *NFKB1*, *RELA*) (Fig. S5C). However, when we cotransfected HEK293 cells with NF-κB luciferase reporter constructs and a construct containing the first exon of *GRA28* (see below), we saw no increase in the levels of luciferase after GRA28 transfection in contrast to a known NF-κB activating construct containing multiple caspase activation and recruitment domains (CARDs) (Fig. 4D). This suggests that NF-κB activation may not play a role in CCL22 induction. Other candidate transcriptional mediators with GRA28-dependent transcript levels are *FOS*, *JUN*, and *IRF4* (Fig. S5D). Transcript levels of *JUN* have been shown in numerous studies to increase in a variety of host cells after infection with T. gondii (23, 24). To test whether GRA28 played a role in altering C-JUN abundance during infection, we infected THP-1 cells with WT or Δ*GRA28* T. gondii parasites for 24 h and using semiquantitative Western blotting to quantify C-JUN protein levels. While infection of THP-1 cells clearly increased C-JUN levels compared to that of mock-treated cells (Fig. S6), the presence or absence of GRA28 in the infecting strain had no significant impact on C-JUN protein abundance. These data suggest that while JUN transcript levels appear to be at least somewhat dependent on GRA28 in the infecting strain (Fig. S5D), this does not appear to be detectable at the protein level using Western blotting.

10.1128/mBio.01591-21.6 FIG S5 Ingenuity pathway analysis of THP-1 cells infected with RH WT or RHΔ*GRA28* T. gondii identifies candidate upstream regulatory gene products that may be driving GRA28-dependent differences in transcript abundance. (A) Genes of higher abundance in RH WT-infected THP-1 cells were found to be significantly associated with multiple immunity-related regulatory genes, including those related to the NF-κB pathway (e.g., NFκBIA, NFκB1, and REL). (B) Hierarchical cluster of correlations in the amount of target gene overlap for each of the upstream regulators shown in panel A. A small cluster of the most highly correlated genes contained multiple genes relevant to NF-κB activation (outlined in green dots). (C) As in panel B, most of the upstream regulators had the same downstream targets, and this was most evident for the AP-1 transcription factor complex (encoded by *FOS* and *JUN* genes) as well as *IL1B* and *ICAM1*. A heat map indicating fold differences between RH WT- and RHΔ*GRA28*-infected THP-1 cells for these downstream targets is also shown. (D) Heat map showing transcript abundance for all transcriptional regulators that were found to be significantly altered in infected THP-1 cells in a GRA28-dependent manner (*P* < 0.001; log_2_ fold difference ≥ 1). Download FIG S5, JPG file, 1.3 MB. Copyright © 2021 Rudzki et al. 2021 Rudzki et al. https://creativecommons.org/licenses/by/4.0/ This content is distributed under the terms of the Creative Commons Attribution 4.0 International license.

10.1128/mBio.01591-21.7 FIG S6 C-JUN protein levels are induced by T. gondii infection in THP-1 cells but do not depend on the presence of GRA28 in the infecting strain. THP-1 cells were infected with the indicated strains (or mock treated by exposing them to the same WT parasite suspensions as for infection but after sterile filtering with a 0.2-μm filter) for 24 h, and then the C-JUN protein level was quantified using Western blotting. Histone H3 levels served as a control, and densitometry was used to calculate the C-JUN/histone H3 ratio as a proxy for normalized C-JUN abundance. Two replicates, with 3 wells of cells for infections and 2 wells for mock treatment, are shown, each having similar results. Download FIG S6, JPG file, 1.3 MB. Copyright © 2021 Rudzki et al. 2021 Rudzki et al. https://creativecommons.org/licenses/by/4.0/ This content is distributed under the terms of the Creative Commons Attribution 4.0 International license.

### The first exon of *GRA28* is sufficient for induction of CCL22 during parasite infection of and ectopic expression in human cells.

**FIG 5:**
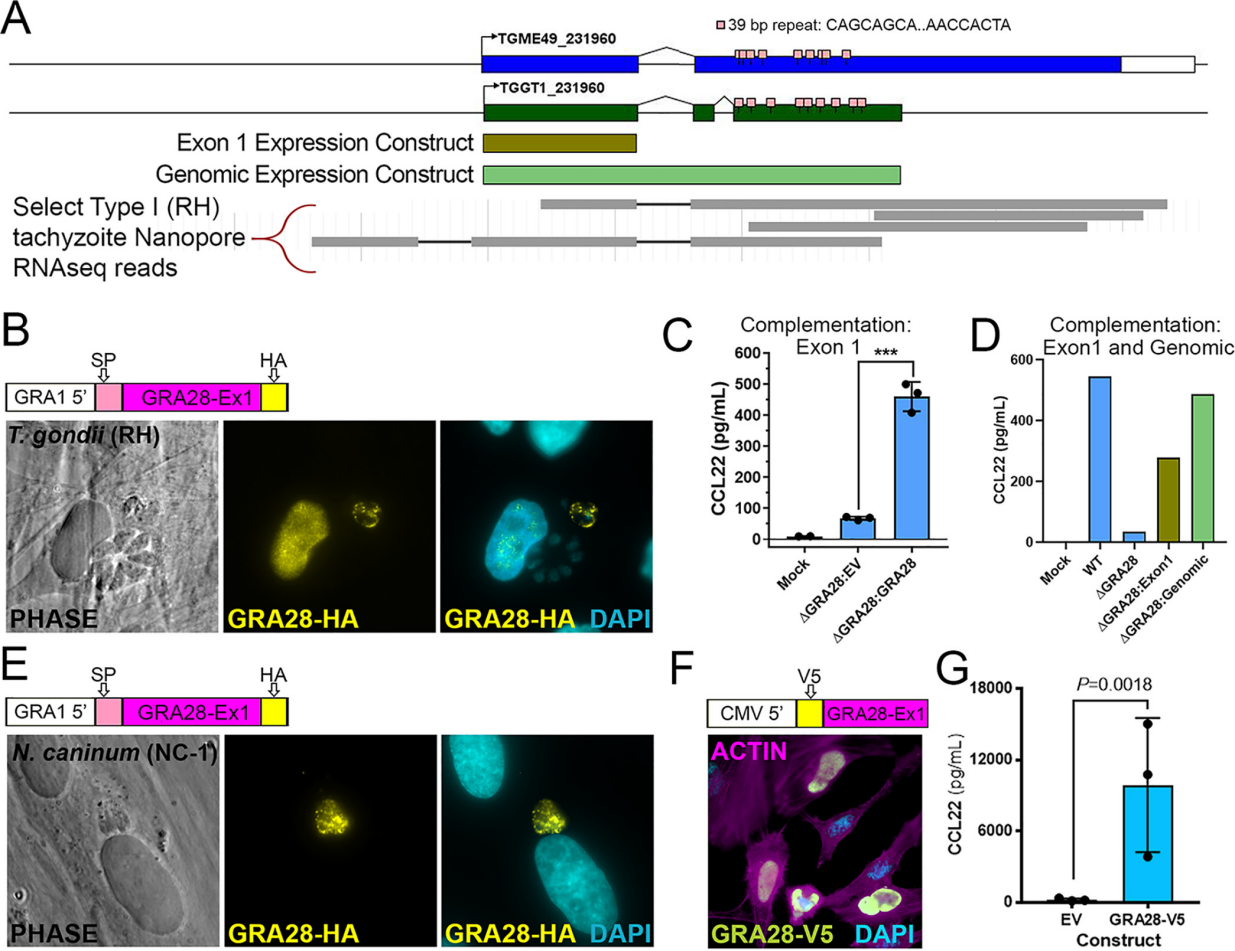
(A) Schematic of the *GRA28* locus along with its gene prediction in the current annotation of the T. gondii genome (https://toxodb.org). In addition to gene models from two T. gondii strains (GT1 and ME49), 39-bp repeats and regions used in expression constructs are shown in brown and light green. The map was created using GenePalette software (see Materials and Methods) (51). (B) Sequence encoding an N-terminal HA tag was inserted immediately after the predicted signal peptide cleavage site in a GRA1-promoter driven version of T. gondii GRA28 exon 1. When transiently transfected into T. gondii, HA-tagged protein could be detected in the parasites as well as the host cell nucleus. (C) Δ*GRA28* T. gondii parasites (RH strain) were transiently transfected with empty pGRA-HA-HPT vector (EV) or the same construct described for panel B encoding an HA-tagged version of T. gondii GRA28 exon 1. After washing in cDMEM, parasites were used to infect freshly plated THP-1 cells for 24 h and CCL22 levels were quantified in culture supernatants using ELISA. Mock-treated cells were exposed to a sterile filtered parasite preparation. (D and E) The construct encoding HA-tagged T. gondii GRA28 exon 1 (same as that used for panels B and C) was used to transfect Neospora caninum, a near relative of T. gondii. HA staining revealed expression of this T. gondii GRA28 exon 1 in N. caninum parasites (visualized by HA staining), but in contrast to T. gondii, we did not observe trafficking of GRA28 to the host cell nucleus when expressed in this strain. Quantification of nuclear HA-derived signal is presented in Fig. S7 in the supplemental material. (F) Sequences encoding an N-terminal V5 tag were inserted downstream of a Kozak consensus sequence and upstream of GRA28 exon 1 (minus the signal peptide-encoding sequence). The construct was transfected into HeLa cells, and V5 staining was observed prominently in the nucleus of transfected cells. (G) Transfection of the construct in panel E directly into RAW 264.7 cells significantly induced CCL22 production, as detected by ELISA. A *t* test was performed on log_10_-transformed data.

The *GRA28* gene has been described previously as encoding a dense granule protein that was capable of trafficking to the host cell nucleus during infection (25). However, the exact structure of the GRA28-encoding gene was somewhat ambiguous based on its annotation in ToxoDB. Specifically, while TGME49_231960 is predicted as a single exon gene spanning ∼7.4 kb of genomic sequence (Fig. 5A), the annotated gene is shorter in TGGT1 (Fig. 5A) and split into two gene products in T. gondii strains VEG, FOU, ARI, VAND, MAS, CATPRC2, and P89. The 5′ end of the gene was consistently predicted across all annotated genes, including the precise location of the first intron. When we performed *de novo* assembly of the T. gondii RH transcriptome, we were unable to identify any assembled transcripts that spanned the entire length of the TGME49_231960 prediction, most likely due to the fact that a 39-bp repeat in between each of these transcripts disrupted the assembly process (repeat consensus sequence, CAGCAGCAGCCACAAGGGWMTGTTGTGCATCAACCACTA) (Fig. 5A). However, it should be noted that when we recently examined released Oxford Nanopore long-read single-molecule sequencing of T. gondii transcripts that are available at https://toxodb.org, there are multiple reads that span this repeat region (select Nanopore reads shown in Fig. 5A), suggesting that the gene is at least similar to that predicted for ME49 in the *Toxoplasma* genome database. Regardless, given the challenges associated with amplifying and cloning this repetitive region, we expressed a hemagglutinin (HA)-tagged version of the first exon of GRA28 in T. gondii and observed expression within both the parasites and the HA signal in the nucleus of infected cells (Fig. 5B). Importantly, CCL22 induction could be restored in an RHΔ*GRA28* clone after bulk transfection of the exon 1 GRA28 expression construct prior to infecting THP-1 cells (Fig. 5C), confirming the role of sequences present in the first exon of GRA28 in driving CCL22 production in human cells. Similar results were obtained when we transiently expressed a construct containing the entire genomic locus for the predicted T. gondii GT1 *GRA28* gene (Fig. 5A, light green bar) in RHΔ*GRA28* parasites (Fig. 5D). In contrast, when the exon 1 construct was expressed transiently in Neospora caninum (strain NC-1) (26), we did not observe any HA signal in the infected host cell despite expression of the protein within the parasite (Fig. 5E). When we quantified host nuclear HA signal intensity (background subtracted and then normalized to staining intensity within the parasite) (see Materials and Methods) in infected host cells, there was a clear and significant (*P = *0.0012) difference in the amount of HA-derived signal in the host nucleus when TgGRA28 was expressed in T. gondii than when it was expressed in N. caninum (Fig. S7A). Close inspection of multiple images suggests that the trafficking of T. gondii GRA28 within N. caninum itself may be distinct from how it traffics in T. gondii. For example, HA staining was observed mostly within the parasite for N. caninum but could be found both within T. gondii and at the vacuole periphery (Fig. 5E; Fig. S7). While N. caninum clearly failed to traffic detectable amounts of GRA28 into the host cell, this could be due to (i) poor trafficking of the protein within the parasite such that it never gains proper access to vacuolar export machinery components like MYR1 and/or (ii) poor trafficking from the parasite into the host cell due to incompatibility with the N. caninum export machinery. Interestingly, N. caninum does not appear to have an intact *GRA28* gene in its genome (see the synteny map for TGME49_231960 at https://toxodB.org), although it does have a *MYR1* ortholog which has been shown to be sufficient to traffic secreted T. gondii proteins into the host nucleus. Finally, GRA28 exon 1 (minus the residues encoding the predicted signal peptide) could be robustly expressed in HeLa cells with a V5 tag, where it trafficked to the host cell nucleus (Fig. 5F), and also was functional when expressed ectopically in THP-1 cells, where it induced CCL22 secretion (Fig. 5F).

10.1128/mBio.01591-21.8 FIG S7 (A) Quantification of nuclear localization in T. gondii (*n* = 5) and N. caninum (*n* = 3) vacuoles expressing exon 1 of T. gondii HA-tagged GRA28. Data were normalized for each image and HA-positive vacuole by subtracting the mean HA intensity in a nucleus neighboring the infected cell from the mean GFP intensity in the nucleus of the infected cell and then dividing that by the mean HA intensity of the parasite-containing vacuole. Expression in T. gondii led to a significantly (*P* < 0.01; *t* test on the log_10_-normalized data) higher normalized intensity in the nucleus than when GRA28 was expressed in N. caninum. (B and C) Schematic illustrating how data in panel A were collected and calculated for T. gondii (B) and N. caninum (C). Download FIG S7, JPG file, 0.5 MB. Copyright © 2021 Rudzki et al. 2021 Rudzki et al. https://creativecommons.org/licenses/by/4.0/ This content is distributed under the terms of the Creative Commons Attribution 4.0 International license.

### GRA28 induction of Ccl22 is fully conserved in mice.

**FIG 6:**
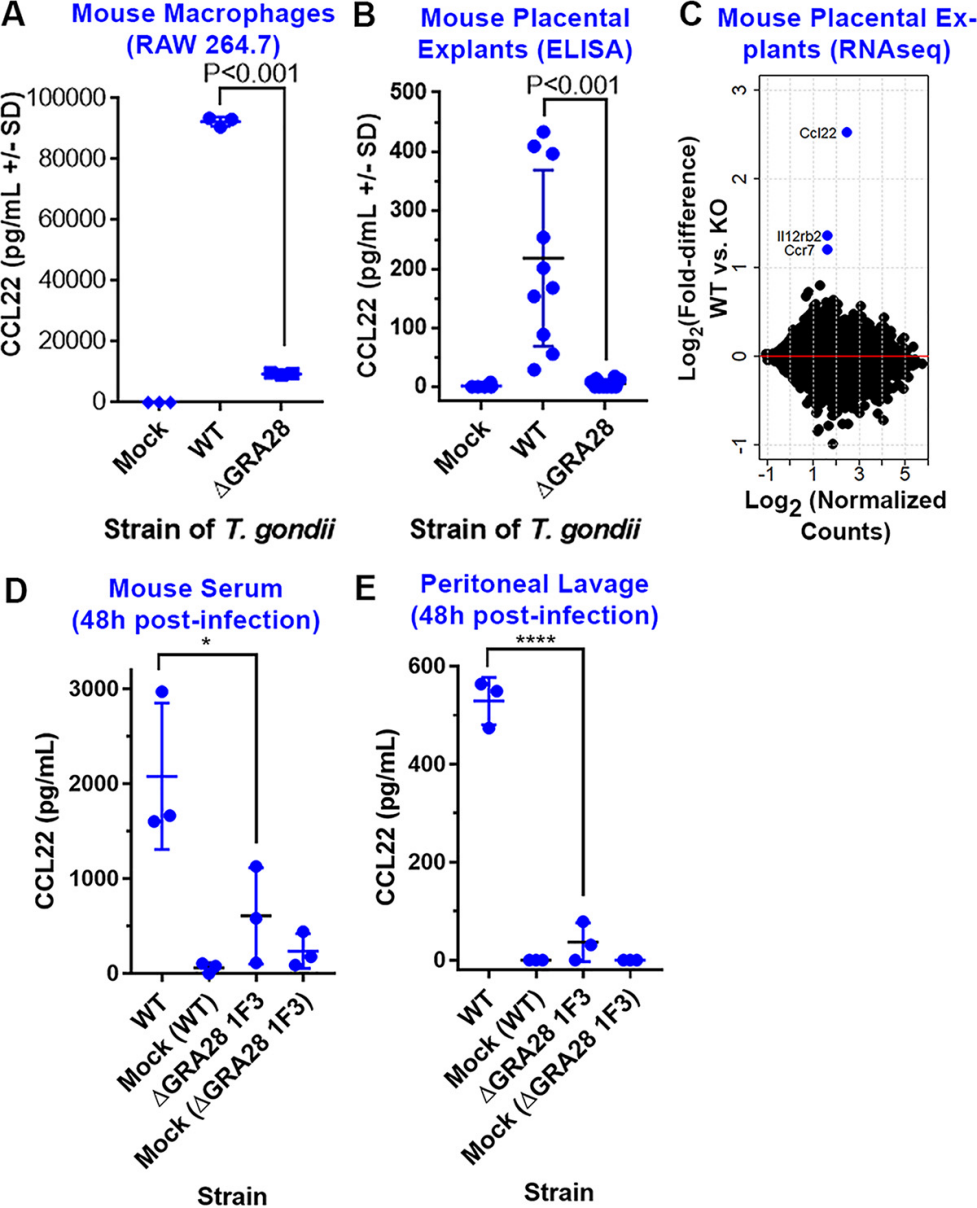
GRA28 induction of Ccl22 is conserved in mouse immune and placental tissues. (A and B) Δ*GRA28* parasites induce significantly less Ccl22 secretion from RAW 264.7 macrophages (A) and mouse placental explants (B) than wild-type parasites. (C) The number of host genes besides Ccl22 that are GRA28 dependent in placental explants is relatively small, suggesting that GRA28 is a highly specific inducer of Ccl22 in mouse placental tissue. (D and E) Mouse serum (D) and peritoneal lavage (E) levels of Ccl22 48 h postinfection are dependent on GRA28.

To determine whether parasite-driven induction of CCL22 is conserved in the murine model, we compared WT and GRA28-deficient (Δ*GRA28*) parasites for their ability to induce this chemokine *in vitro*, *ex vivo*, and *in vivo*. First, we infected mouse macrophages (RAW 264.7) *in vitro* with type 1 strain (RH) T. gondii parasites (WT) or RHΔ*GRA28* T. gondii parasites at MOIs of 3. Based on Ccl22 ELISA, mouse macrophages not only release more Ccl22 protein during T. gondii infection, but similar to human THP-1 cells, this phenotype is also dependent on the presence of T. gondii secreted protein GRA28 (Fig. 6A). Next, we investigated whether primary mouse tissues, specifically mouse placental tissue, also elicit this response to T. gondii infection. Embryonic day 12.5 Swiss Webster mouse placentas were halved and distributed into separate treatment groups. These placental explants were then infected *ex vivo* with 2.0 × 10^6^ type 1 strain (RH) T. gondii parasites (WT), RHΔ*GRA28* T. gondii parasites, or mock treatment. As shown in Fig. 6B, primary mouse placental tissue also responds to T. gondii infection by releasing Ccl22 protein in a GRA28-dependent manner. RNA was also extracted from the infected placental samples, and we performed RNA-seq. As shown in Fig. 6C, the number of transcripts that varied in a GRA28-dependent manner was markedly small, suggesting that GRA28 is a highly specific inducer of *Ccl22* in mouse placental explants. Of the three genes with significantly higher transcript levels (*Ccl22*, *Il12rb2*, *Ccr7*) in wild-type 1 infections compared to RHΔ*GRA28* infections, *Ccl22* was the most highly induced. These data show conservation of the parasite-driven Ccl22 phenotype in primary mouse placental explants at both a protein and transcript level. Finally, we investigated mouse *in vivo* Ccl22 responses to T. gondii intraperitoneal infection. Female BALB/cJ mice (*n* = 3 for each treatment) were infected with WT, Δ*GRA28*, or mock T. gondii treatments. We focused on early, acute infection and performed Ccl22 ELISA on serum (Fig. 6D) and peritoneal lavage fluid (Fig. 6E). These suggest that *in vivo* Ccl22 protein levels are at least partially dependent on GRA28. Moreover, while there was a significant amount of systemic Ccl22 protein detected in serum of infected mice, even in the Δ*GRA28* parasite treatment, Ccl22 was almost undetectable in peritoneal lavage fluid in RHΔ*GRA28*-infected mice. Overall, these data indicate that the process driving T. gondii GRA28-induced Ccl22 is similar, if not the same, in both mice and humans and that this parasite effector can mediate robust changes in Ccl22 production at the site of infection and systemically.

### GRA28-deficient parasites have distinct inflammatory and dissemination phenotypes in the acute and chronic phases of infection, respectively.

**FIG 7:**
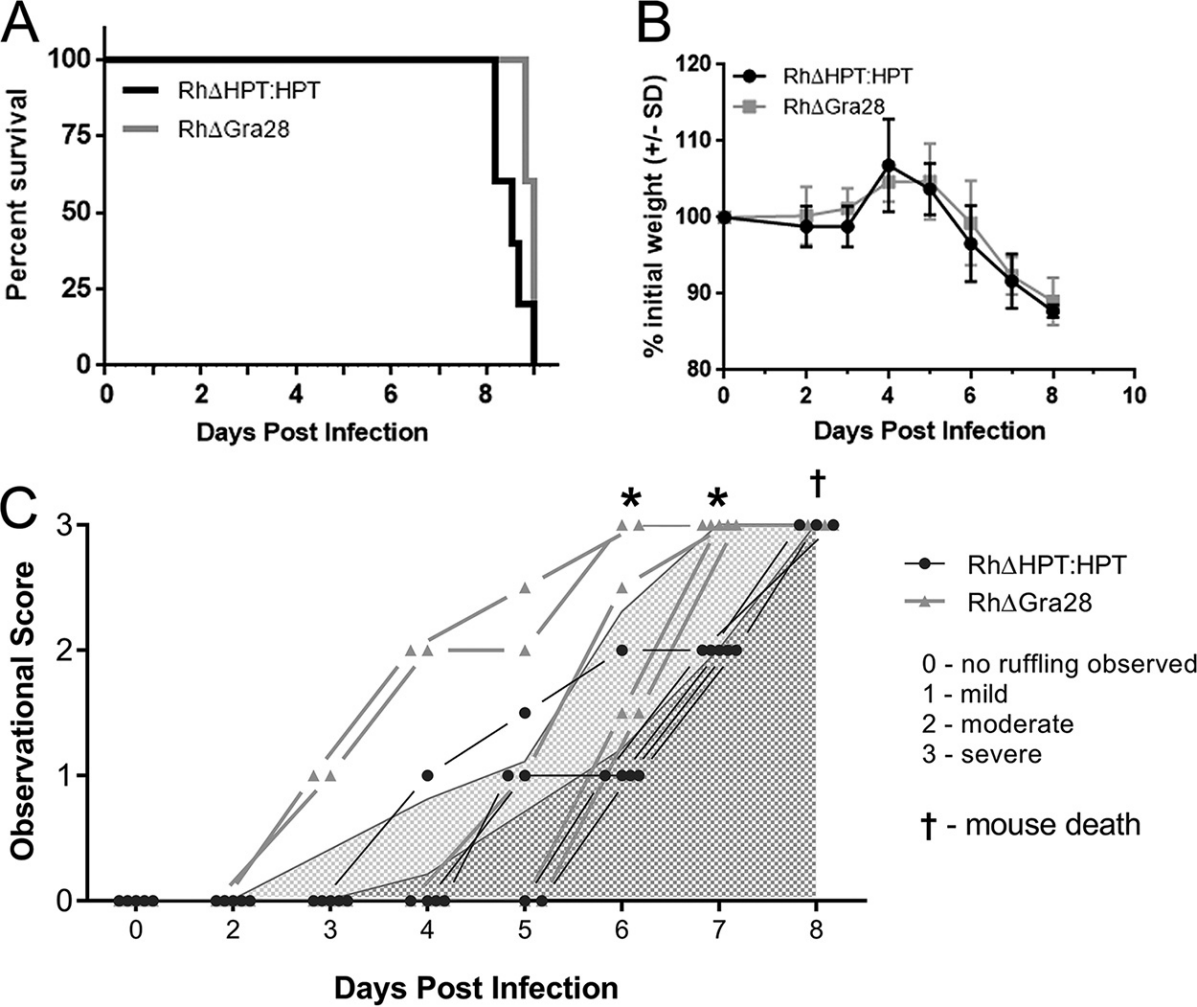
(A and B) Mortality (A) and weight loss (B) does not significantly differ in mice infected with WT and Δ*GRA28* type 1 strain T. gondii parasites. (C) Behavioral changes and fur ruffling phenotypes associated with infection are exacerbated during the acute phase of infection for mice infected with Δ*GRA28* parasites compared to that in WT parasites based on a phenotype scoring system. Specifically, mice infected with Δ*GRA28* parasites (light gray lines and triangles) exhibited infection-related symptoms at earlier time points than those infected with WT parasites (black lines and circles). Curves beneath lines are the average across all mice at that time point (with lighter gray representing Δ*GRA28* knockout parasites and darker gray representing the wild type). *, *P* < 0.05 after two-way ANOVA and followed by multiple comparisons at each time point.

To determine the impact of T. gondii GRA28 *in vivo*, we indexed differences in mouse behavior relevant to inflammatory responses and quantified differences in infection-induced weight loss and total morbidity after infection of BALB/cJ mice with either RH WT or RHΔ*GRA28* T. gondii. We observed no significant differences in morbidity or weight loss (Fig. 7A and B). However, when we scored (Fig. S8) mice over the course of infection as to the extent of inflammation-induced behavioral changes, we observed significantly heightened fur ruffling in the RHΔ*GRA28*-infected mice on days 6 and 7 postinfection (Fig. 7C), despite the fact that mortality was unchanged.

10.1128/mBio.01591-21.9 FIG S8 Visual representation of pathology index scores. All images are the same individual from two viewpoints (lateral and dorsal) at four different timepoints of infection. Scores: 0, no fur ruffling or red/irritated skin present; 1, mild ruffling present, located predominantly on the head and back of the neck; no red/irritated skin visible; 2, moderate ruffling present—fur forms larger clumps and extends to the rest of the body; skin may be visible through the clumps but is not red or irritated; 3, severe ruffling is characterized by ruffling across the entire body with visibly red/irritated skin. Download FIG S8, TIF file, 1.6 MB. Copyright © 2021 Rudzki et al. 2021 Rudzki et al. https://creativecommons.org/licenses/by/4.0/ This content is distributed under the terms of the Creative Commons Attribution 4.0 International license.

**FIG 8:**
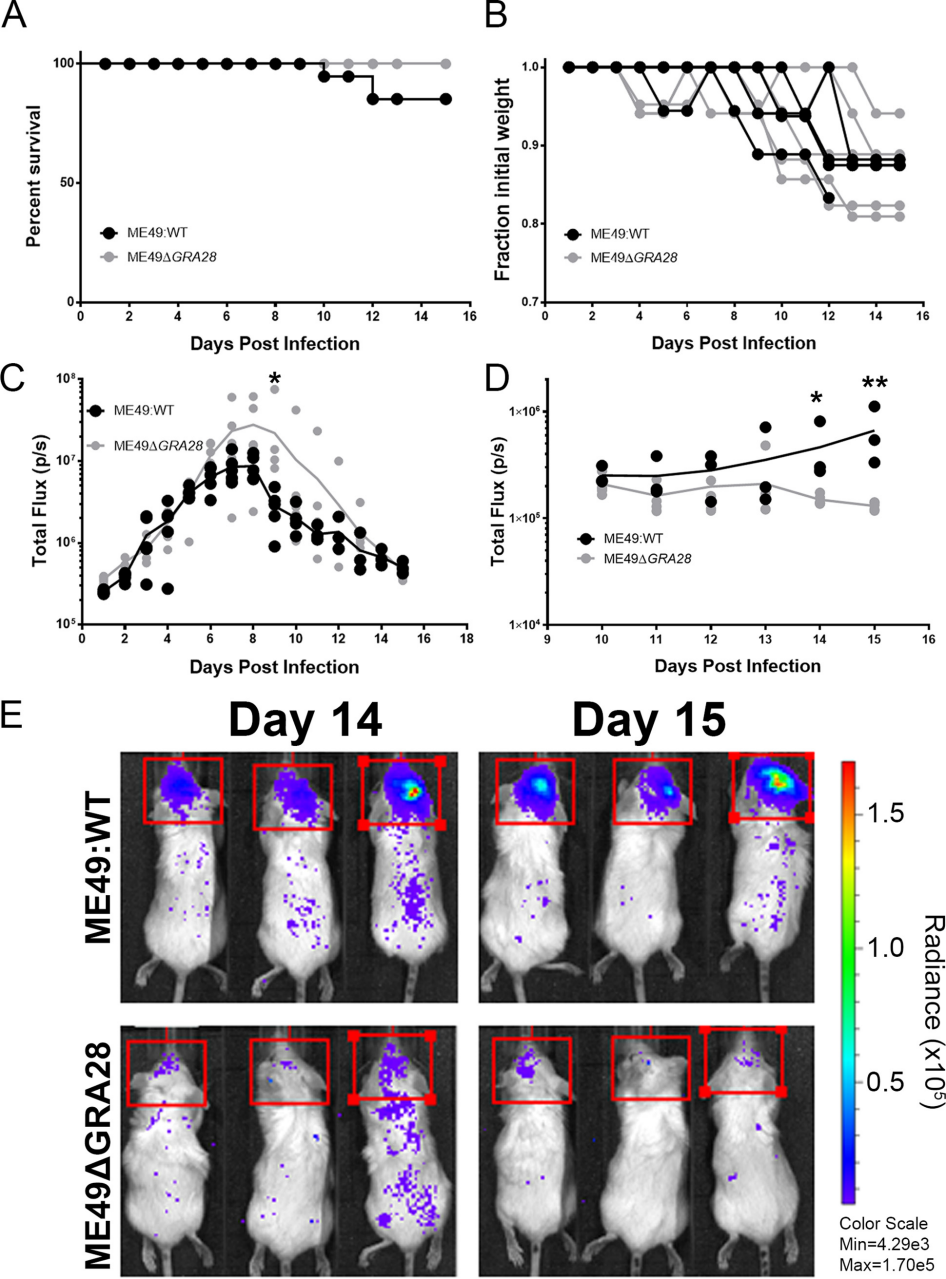
Impact of the *GRA28* gene on proliferation and dissemination of type 2 parasites expressing luciferase. (A and B) Neither mortality (A) nor mouse weight loss (B) was significantly different in mice infected with WT or Δ*GRA28* T. gondii type 2 (ME49) strain parasites. (C) Throughout the acute phase of infection, parasite-derived bioluminescence of WT and Δ*GRA28* parasites in the peritoneal cavity was measured by imaging the animals ventrally. The burdens were similar (*P* > 0.05) between parasite strains at all time points except for day 9 postinfection, when the signal was significantly higher in Δ*GRA28*-infected mice than in WT mice (*P = *0.014). (D and E) Starting on day 10 postinfection, we imaged mice both ventrally and dorsally to visualize dissemination to and proliferation in the mouse brain. In contrast to findings for the acute phase, we observed a consistently lower level of parasite-derived bioluminescence in the brains of mice infected with Δ*GRA28* parasites than with WT parasites on days 14 and 15 postinfection (*P = *0.016 and 0.0002, respectively). All statistical tests were performed on log_2_-transformed bioluminescent data.

We also generated Δ*GRA28* parasites in a type 2 T. gondii background that had been previously engineered to express luciferase and green fluorescent protein (GFP) (specifically ME49Δ*HPT:LUC* [27, 28]) to permit noninvasive quantification of parasite burden and dissemination over the course of infection. For the ME49 strain infections, we observed only minor and nonsignificant differences in mouse morbidity and weight loss (Fig. 8A and B). However, during the acute phase of infection, we observed slight differences in parasite burden between ME49Δ*HPT:LUC* (WT) and ME49Δ*GRA28*-infected mice, with the burden being significantly higher in ME49Δ*GRA28* than in WT mice on day 9 postinfection (Fig. 8C). This difference was not due to experimental variation in parasite input between strains, since the parasite burden was indistinguishable during the first 6 days postinfection (Fig. 8C). In contrast to these minor differences during the acute phase of infection, we observed more dramatic differences in parasite burden during the later stages of infection. Specifically, quantification of *in vivo* bioluminescence data taken dorsally on days 14 and 15 postinfection revealed that WT parasites were of much greater abundance in the brains than those infected with ME49Δ*GRA28* (Fig. 8D and E).

## DISCUSSION

T. gondii-infected host cells have dramatically altered transcriptomes compared to uninfected cells, and effectors that are secreted from the parasite during invasion drive most, but not all, of these changes (29). To date, the vast majority of these parasite effectors are derived from the dense granule and rhoptry organelles. We previously identified that T. gondii induces the production of CCL22 in human placental trophoblasts, while human foreskin fibroblasts do not exhibit this chemokine induction during T. gondii infection (4). Additionally, our previous work has shown that this induction required parasite invasion and the effector chaperone-like T. gondii gene product MYR1 (4, 13). While T. gondii induces CCL22 during infection of a variety of cell types from both mice and humans (18, 30), including at the transcriptional level in mouse brain (31), placental cell CCL22 induction is driven by a highly specific parasite effector, GRA28. CCL22 production is considered to be an indication of M2 macrophage polarization, and macrophage polarization has been linked to strain-specific T. gondii effectors like ROP16 and GRA15 (32). The impact of GRA28 is distinct from these effectors because CCL22 induction occurs similarly in all three canonical strain types, and GRA28 does not alter the expression of other M2-associated genes (such as *IL-4*, *IL-10*, *IL-13*, or *ARG1*) in the cell types that we have assayed. The specificity of GRA28 for only a few target genes is novel compared to effectors like GRA15 and ROP16 (24, 33) that alter the abundance of hundreds of transcripts.

Transcriptional coregulation has been used in other systems as a means to identify members of protein complexes (34), but to our knowledge, this is the first time this approach has been successfully applied at this scale in T. gondii. We used 396 microarray data sets derived from multiple T. gondii life stages and experimental manipulations to provide enough variation to better distinguish subclusters within closely related gene families. Genes encoding dense granule proteins are among the most highly expressed in the T. gondii genome, making them more difficult to separate from one another, but they still clustered into two distinct groups with functional themes. The MYR1/GRA28 cluster harbored a handful of known secreted dense granule effectors, while the other contained genes encoding dense granule structural proteins or those that are secreted into the vacuole but do not traffic to the host cell. We anticipate that the former cluster can be exploited further to identify additional MYR1-trafficked, and putatively host-modulating, effectors, while the latter has highlighted new candidates important in dense granule structure or function within the parasite. The entire data set is available for download as a text file at Figshare (https://doi.org/10.6084/m9.figshare.16451832) so that these data can be mined to identify candidates for membership in other critical T. gondii-specific protein complexes.

*GRA28* was previously shown to encode a dense granule protein secreted from the parasite into the host cell, where it trafficked to the host cell nucleus (25), but its impact on the host cell was unknown. Its natural presence in the host nucleus during infection has also been further confirmed using proteomics, where it was found to be one of the more abundant T. gondii proteins in the nucleus of the infected host cell (35). The fact that it affects the abundance of only a small number of chemokine-encoding genes at the transcriptional level suggests that it modulates transcriptional activity via direct interactions with transcription factors and/or upstream regulatory sequences. Other T. gondii effectors traffic to the host nucleus, but this is not always critical for function. For example, ROP16 localization to the host cell nucleus is dispensable for its primary function of phosphorylating STAT6, which occurs in the cytoplasm of the host cell (36). Other T. gondii effectors like IST (15, 37) and GRA24 (16) function within the host cell nucleus, but many of these mediate changes in hundreds of transcripts via their cooperation with existing transcriptional suppressors (IST [15, 37]) or activators (GRA24 [16]). It remains to be seen if the function of GRA28 can occur independently of nuclear trafficking or if this ultimate localization is required for chemokine induction, but its specificity for downstream genes raises the interesting hypothesis that it may function directly, possibly as a heterologous transcription factor.

The signaling pathway governing GRA28 function is unknown, but some clues can be found in our pathway analyses, which suggest a role for GRA28 in mediating changes in key immunity-related host cell signaling pathways. The transcription factor genes *JUN*, *FOS*, and components of the NF-κB complex were consistently linked to the GRA28-dependent host transcripts. Lipopolysaccharide (LPS) is a well-known activator of both NF-κB and C-Jun activity in THP-1 cells (38, 39), and this can occur via Toll-like receptor activation (40). However, T. gondii induction of CCL22 was not fully dependent upon host MYD88, since MYD88^(−/−)^ THP-1 cells still produced significant amounts of CCL22 in response to T. gondii infection (see Fig. S1C in the supplemental material). The difference in CCL22 production by the MYD88^(−/−)^ cells in comparison to the WT cells should also be considered in light of the fact that the cell lines have different origins (and therefore distinct passage histories, which could have the more subtle effects shown on CCL22 production after infection). A distinct cluster of GRA28-dependent host genes was identified that encoded gene products involved in proteoglycan synthesis, including the rate-limiting enzyme XYLT1. T. gondii attachment to host cells is mediated by interactions between parasite adhesins and host cell surface sulfated proteoglycans (PG) like heparan sulfate (41, 42), and T. gondii adheres poorly to cells with genetically or enzymatically depleted levels of surface sulfated proteoglycans (41, 42). Therefore, direct and/or indirect modulation of XYLT1 transcript levels by GRA28 may serve to make infected cells susceptible to adhesion, and ultimately invasion, by T. gondii or any other pathogens that depend on surface proteoglycans.

GRA28 had no impact on transcript levels of the gene encoding CCL17, which is commonly coregulated with *CCL22*. Mouse macrophages infected with T. gondii produce Ccl17 and Ccl22, and this is due, at least in part, to another T. gondii effector, GRA18 (18). Using the same *GRA18* knockout lines (kindly provided by the Bougdour lab), we found that GRA18 had no impact on CCL22 production at the transcriptional (not shown) or protein (Fig. 4) level in human THP-1 cells, suggesting that GRA18 and GRA28 have distinct targets. This is also consistent with the observation that Ccl22 induction in RAW macrophages is only partially dependent on GRA18 and β-catenin signaling, in contrast to Ccl17 and Ccl24. Finally, in our work, we used lower MOIs (2 to 3 here compared to 5 to 6 in reference 18). Regardless, GRA28 appears to be the more potent modulator of Ccl22 production compared to GRA18, while Ccl17 appears to be much more dependent on GRA18. It is exciting to speculate that T. gondii *GRA28* has evolved to uniquely target CCL22 as a means to gain access to the fetal compartment, since this chemokine is potently induced in placental cells and this chemokine plays a role in immune tolerance during pregnancy (7). However, as shown clearly in this study, GRA28 also alters monocyte/macrophage CCL22 production, making it equally plausible that this intricate molecular relationship developed first as a more generalized immune evasion (via suppression) strategy.

The role of specific chemokines like CCL22 during T. gondii infection is poorly understood, but the discovery of *GRA28* allows this to be addressed more directly using T. gondii Δ*GRA28* parasites from different genetic backgrounds. Hypervirulent T. gondii RH strain Δ*GRA28* parasites caused inflammation-related behavioral changes earlier during infection in mice in comparison to mice infected with WT parasites, suggesting that GRA28 functions to suppress inflammatory responses (likely due to induction of CCL22, although we did not test this directly). This could arise via GRA28-mediated recruitment and/or activation of regulatory T cells to the site of infection. These behavioral changes occurred without an effect on the acute virulence phenotype, as all mice succumbed to the infection with similar kinetics, which is consistent with an impact of GRA28 on suppressing inflammatory responses without altering the ability of the mouse to control parasite replication. However, after infections using the type 1 parasite genotype, we observed a significant reduction in Δ*GRA28* parasite burden in the brain compared to that of wild-type parasites. This effect was unexpected, given the fact that the parasite burdens were statistically the same during the acute phase of infection, but points to a potential important role for GRA28 in altering the host innate immune response in a manner that increases host susceptibility to dissemination of T. gondii across critical barriers like that guarding the central nervous system (CNS). T. gondii can infect blood-brain barrier epithelial cells as a means to cross into the host CNS (43), so GRA28 may promote parasite survival at this critical interface by recruitment of regulatory T cells or other cell types that might downregulate inflammatory responses.

### Summary.

Taken together, our data point to a specific role of T. gondii GRA28 in modulating chemokine production in the infected cell. Importantly, this effect occurs only in certain cell types, including cells from both human and mouse placenta. A relatively small number of host chemokines are affected by parasites expressing this gene, and it plays a role in both modulation of the inflammatory response (as evidenced by mouse behavior and appearance during infection) and ultimately parasite dissemination to “privileged” sites like the CNS.

## MATERIALS AND METHODS

### Cell culture.

All cell and tissue cultures were incubated at 37°C and 5% CO_2_. All media were supplemented with 10% fetal bovine serum (FBS; Atlas Biologicals), 2 mM l-glutamine, and 50 mg/ml penicillin-streptomycin. Human foreskin fibroblast (HFF) cells were grown in Dulbecco’s modified Eagle medium (DMEM; Gibco), Raw264.7 cells were grown in DMEM (Gibco) with 10 mM HEPES, and THP-1 cells were grown in RPMI 1640 medium (Corning). THP-1 cells were assayed for viability using trypan blue staining (0.4%) (Gibco), counted, and spun at 120 × *g* for 10 min at 24°C, and the medium was replaced with supplemented DMEM prior to infection. All THP-1 cell numbers listed are based on trypan blue-negative cells. Media containing serum, glutamine, and antibiotics are called “complete” and have a prefix of “c” in the text (e.g., cDMEM).

### Human placental explants.

Human placental tissue from less than 24 weeks of gestation was obtained, cultured, and infected with T. gondii as described previously (44).

### Mouse placental explants.

Mouse placental tissues were obtained by dissection of embryonic day 12.5 (E12.5) or E18.5 Swiss Webster mice. Upon removal of the fetuses from the mother, the placentas were dissected away from other tissues and placed into prewarmed 37°C phosphate-buffered saline (PBS). The placentas were washed three times in fresh prewarmed PBS. Each placenta was then cut in half with sterilized surgical scissors, and each half was placed into a well on a plate with prewarmed 37°C DMEM with 10 mM HEPES, 10% FBS, 2 mM l-glutamine, and 50 mg/ml penicillin-streptomycin. Each placenta had one half-piece of tissue represented in each treatment group. For T. gondii infections, isolated tissue was infected immediately with 5.0 × 10^5^ to 2.0 × 10^6^ parasites for ∼24 h.

### Parasites.

Type 1 (RH, GT1), type 2 (Me49, PRU), and type 3 (Veg, CEP) Toxoplasma gondii tachyzoites and sporozoites, Neospora caninum (NC-1) tachyzoites, and Hammondia hammondi (HhCatAmer and HhCatEth1 [45, 46]) sporozoites were used in this study. Sporozoites were excysted from sporulated oocysts as described previously (47) and either used immediately or grown for 24 h in HFFs prior to being used in controlled infections. Tachyzoites were maintained by continual passage in HFF cultures incubated at 37°C and 5% CO_2_ in DMEM supplemented with 10% FBS (Atlas Biologicals), 2 mM l-glutamine, and 50 mg/ml penicillin-streptomycin. The RH*YFP* strain was a gift from David Roos (University of Pennsylvania), the RHΔ*MYR1* and RHΔ*MYR1:MYR1_c_* parasites (13) were a gift from John Boothroyd (Stanford University), the PRUΔGRA18 and complemented knockout parasites were shared by Alexandre Bougdour (18), and the PRUΔ*Toxofilin* KO parasites (19) were a gift from Melissa Lodoen (UC Irvine). For infections, infected monolayers were washed with fresh cDMEM and then scraped and syringe lysed to release tachyzoites. These tachyzoites were then passed through a 5-μm syringe filter and counted. Parasites were then centrifuged at 800 × *g* for 10 min at 24°C and resuspended and diluted in cDMEM before being used in infections. Mock treatments were produced by filtering the same parasites through a 0.22-μm syringe filter and exposing host cells to the same volume of the filtrate as was used for parasite infections. Freeze treatments were produced by subjecting the parasites to −80°C for 15 min, fixation treatments were produced by exposure to 4% paraformaldehyde for 10 min followed by washing in PBS, and sonication treatments were produced by sonication at 0°C using five 30-s bursts at 50 A with 30-s cooling intervals between bursts, followed by microcentrifugation at 800 × *g* for 10 min to generate soluble (S) and pellet (P) fractions.

### Invasion inhibitor assays.

For cytochalasin D (Cyt-D) treatment, parasites were pretreated with 10 μg/ml of Cyt-D in cDMEM for 1 h and then used to infect cells in the presence of 10 μg/ml of Cyt-D in cDMEM for the duration of infection. Vehicle of Cyt-D is dimethyl sulfoxide (DMSO) (40 μl per ml of cDMEM). For 4-bromophenacyl bromide (4-BPB) treatment, parasites were pretreated with either 0.5 or 1 μM 4-BPB for 15 min. 4-BPB was dissolved directly in cDMEM. Parasites were then washed twice with normal cDMEM with 10-min 800 × *g* spin steps between each wash and then used to infect cells in the presence of normal cDMEM.

### Plaque assays.

Parasites were serially diluted in medium and used to infect monolayers so that each tissue culture flask of HFFs received 100 parasites. These flasks were then incubated at 37°C in 5% CO_2_ undisturbed for 5 to 7 days. At the end of the incubation period, each flask was counted for number of plaques present and parasite viability was calculated. Crystal violet staining was used to count plaques as follows: the monolayer was washed with PBS and fixed for 5 min with ethanol. Then, crystal violet solution (12.5 g crystal violet in 125 ml ethanol mixed with 500 ml 1% ammonium oxalate in water) was introduced to the monolayer and allowed to stain for 5 min. The monolayer was then washed extensively with PBS and allowed to air dry prior to the counting of plaques.

### Candidate gene identification using transcript level correlation analysis.

To identify candidate effectors for inducing CCL22, we exploited the fact that the CCL22 induction response in THP-1 and placental cells required the presence of the T. gondii effector translocation protein MYR1 (13, 14). We hypothesized that MYR1-dependent effectors would have similar transcript abundance profiles across diverse expression data sets. We downloaded 396 publicly available T. gondii microarray expression data sets from the Gene Expression Omnibus platform hosted by the NCBI (48). We loaded and processed each CEL file using the “affy” module implemented in R (49). Data were processed and normalized using the following commands: bgcorrect.method = “rma,” normalize.method = “quantiles,” pmcorrect.method = “pmonly,” summary.method = “medianpolish”. RMA-normalized data were exported, reimported into R, and then transposed. An all-versus-all Pearson correlation matrix was generated using the “cor” function from the R:Stats base module. Probe names on the Affymetrix array (in the format of XX.mXXXXXX) were converted to current TGME49 gene models using data downloaded from ToxoDB and the Vlookup function in Microsoft Excel. In some cases, the microarray annotations could not be matched to current TGME49 gene model names and are shown as blanks in plots. This correlation matrix, the normalized array data used to generate it, and a key to convert Affymetrix probe names to current gene model names are all available on Figshare (https://doi.org/10.6084/m9.figshare.16451832). To analyze this correlation matrix, we used hierarchical clustering tools implemented in R, including heatmap.2 (from the gplots package) and the dendextend package. To identify candidate genes using this matrix, we calculated the mean correlations between 5 bait genes and all other queried genes from the microarray. The bait genes were known to encode either components of the MYR complex themselves or known TgMYR substrates (TgMYR1 [13], TgMYR2 and TgMYR3 [14], TgGRA24 [16], and TgIST [15]). Most of the candidate CCL22-inducing genes were identified based on having (i) an average correlation with the 5 above-listed “bait” genes of ≥0.7, (ii) a *dN*/*dS* ratio of ≥2, and (iii) the presence of a predicted signal peptide or at least one transmembrane domain (which we reasoned could be a cryptic signal peptide if the wrong start codon was chosen for the current gene annotation).

### *De novo* transcript assembly.

To identify and assemble transcripts coding for GRA28, we used the *de novo* transcript assembler Trinity (50) (version 2.6.6; default settings) using triplicate RNA-seq data sets from WT T. gondii RH parasites infecting THP-1 cells (see below). Assembled transcripts with similarity to the predicted *GRA28* gene (TGME49_231960) were identified using BLASTN and BLASTX. Primary plots were generated using GenePalette software (51) and then modified.

### CRISPR-mediated gene disruption and validation of knockouts.

The pSAG1::CAS9-U6::sgUPRT plasmid provided by David Sibley (Addgene plasmid no. 54467 [52]) was modified using the Q5 site-directed mutagenesis kit (NEB) so that the guide RNA (gRNA) sequence was replaced with two restriction enzyme sites (sequence, GTTTAAACGGCCGGCC) for PseI (NEB R0560S) and FseI (NEB R0588S). This modified plasmid was then used as the template for all future Q5 reactions. Two unique gRNA sequences were created for each candidate gene by utilizing the genomic sequences for T. gondii GT1 (https://toxodb.org) and E-CRISP (www.e-crisp.org) using the ToxoDB-7.1.31 reference genome. A forward primer for each gRNA was created for use with the modified pSAG plasmid, with the unique gRNA sequence followed by a section of the plasmid scaffolding (GTTTTAGAGCTAGAAATAGCAAG). The reverse primer used with this plasmid is AACTTGACATCCCCATTTAC. The gRNA sequences for the genes mentioned in this study and the primers used to validate the knockouts are listed in Table S1 in the supplemental material.

A plasmid was created for each gene of interest (GOI) using the modified pSAG plasmid template and the Q5 site-directed mutagenesis kit (NEB) by following the manufacturer’s protocol with a few adaptations. The KLD enzyme step was extended to 60 min of incubation at room temperature, and following the KLD enzyme step, the product was heated to 65°C for 20 min and then double digested with PseI and FseI in CutSmart buffer (NEB) for 60 min at 37°C to remove any remaining plasmid that was not eliminated by the DpnI in the KLD step. This digested product was then heated to 65°C for 20 min to deactivate the enzymes prior to transformation, plasmid isolation, and sequencing to validate insertion of the correct gRNA sequence (pSAG:GOI:gRNA). Parasites were transfected with either a single gRNA plasmid or equal amounts of plasmids encoding two gRNAs targeting the same gene. For validation of knockouts after cloning, a clone was considered a knockout if PCR across a targeted cut site failed or if a PCR across the entire gene (just upstream and downstream of the start and stop codons, respectively) failed. In some cases where all PCRs worked (indicating that the plasmid failed to insert at the gRNA target site), the amplified band was sequenced and a clone was considered a knockout if insertions/deletions were identified near the gRNA binding that resulted in frameshifts and premature stop codons. Validation data for all knockout strains used except the T. gondii Δ*Toxofilin* parasites (which were provided by Melissa Lodoen, University of California-Irvine) are shown in Fig. S4.

### Parasite transfections.

In general, transfections were performed using standard approaches. Briefly, parasite suspensions were obtained by needle passage (25- and 27-gauge needles) and then pelleted for 10 min at 800 × *g*. Parasites (∼2 × 10^7^ per transfection) were resuspended in Cytomix (120 mM KCl, 0.15 mM CaCl_2_, 10 mM KPO_4_, 25 mM HEPES, 2 mM EDTA, 5 mM MgCl_2_, pH 7.6) containing glutathione (GSH) and ATP and electroporated at 1.6 kV with a capacitance setting of 25 μF using a BTX ECM600 electroporator. Transfected parasites were then used to infect coverslips and/or flasks of confluent HFFs and placed under appropriate selection. For candidate gene knockouts, ∼2 × 10^7^ T. gondii RHΔ*HPT* parasites were transfected with ∼30 to 50 μg of the relevant pSAG:GOI:gRNA plasmid(s) (described above) along with 2 to 5 μg of an empty pGRA-HA-HPT (53) plasmid. Parasites were placed under selection the next day and cloned by limiting dilution after 2 to 3 passages. Individual clones were screened for gene deletion by PCR and sequencing to permit identification of both target gene disruptions (via insertion of the pGRA-HA-HPT plasmid at the CAS9 cut site) or mutation via DNA repair events at the CAS9 cut site. For HA-tagging experiments, type 1 (RH) *GRA28* exon 1 (residues 1 to 498) was C-terminally HA tagged by cloning into the T. gondii expression plasmid pGRA-HA-HPT (53). This plasmid drives protein expression using the highly active *GRA1* promoter. TgRHΔ*HPT* or N. caninum Liverpool (NcLIVΔ*HPT*) (54) parasites were transfected with ∼40 to 60 μg of *GRA28* exon 1 plasmid, and cells were grown overnight in normal medium. For analysis of transiently transfected parasites, cells were only grown for 18 h posttransfection, while for stable transfection, parasites were grown for 2 to 3 passages in medium containing 50 μg/ml of mycophenolic acid and xanthine. Cells were fixed with 4% paraformaldehyde (PFA) and permeabilized in 0.1% Triton–PBS. Samples were probed with anti-HA rat monoclonal antibody (3F10 clone; Roche) diluted to 0.1 mg/ml in 0.1% Triton–PBS buffer and washed four times in PBS. Samples were then incubated in 488 goat anti-rat antibody (Life Technologies, Alexa Fluor H+L), followed by PBS washes. All samples were mounted in Vectashield with DAPI (4′,6-diamidino-2-phenylindole) (Vector Laboratories). For genetic complementation, TgRHΔ*GRA28* parasites were transfected with the exon 1 construct described above or a construct amplified from genomic DNA encompassing the start and stop codons of the GT1 version of GRA28 (TGGT1_231960). Expression plasmids (∼30 μg) were cotransfected along ∼5 μg of pLIC_3×HA_DHFR* plasmid (kindly provided by Vern Carruthers) (55), and populations were placed under 1 μM pyrimethamine selection for 2 to 3 weeks and then used to infect THP-1 cells for 24 h as described above, followed by assays to quantify CCL22 in culture supernatants by ELISA.

### Fluorescence image analysis.

To compare signal intensities in the nucleus of host cells infected with either T. gondii or N. caninum parasites transiently transfected with the HA-tagged GRA28 exon 1 construct, we scanned stained coverslips for GRA28-HA-positive vacuoles and then used Fiji (an implementation of ImageJ) to calculate (i) the average HA signal intensity in the nucleus of the infected host cell (*AvgIntInf*), (ii) the average HA signal intensity in the nucleus of a neighboring, uninfected host cell (*AvgIntUninf*), and (iii) the average signal intensity of the parasite-containing vacuole (*AvgIntVacuole*). We then used the following calculation to determine the normalized, background-subtracted nuclear signaling intensity:

$$\frac{\left(\mathrm{AvgIntInf}-\mathrm{AvgIntUninf}\right)}{\mathrm{AvgIntVacuole}}$$

Example images of this process are shown in Fig. 5 and in Fig. S7 in the supplemental material. Data were log_10_ transformed prior to performing a Student’s *t* test.

### RNA-seq.

RNA was isolated from cultures using the RNeasy minikit (Qiagen) and its associated RNase-free DNase digestion set (Qiagen), in accordance with the manufacturer’s protocol for mammalian cells. An Agilent bioanalyzer was used to check the quality of the RNA samples. Tru-Seq stranded mRNA libraries were generated from 5 to 17 ng/μl of mRNA for THP-1 cells, and from 50 to 120 ng/μl of mRNA for murine peritoneal exudate cells (PECs) and placental explants, and sequenced with an Illumina NextSeq 500 sequencer. mRNA-Seq FASTQ reads were mapped to the human reference genome (Homo sapiens v81; hg38) using default options on CLC Genomics Workbench 11 (Qiagen). Total gene reads (with at least 1 read count) were exported from CLC Genomics Workbench and used for DESeq2 (56) to perform differential expression analysis using methods outlined previously (for an example, see reference 4). Data were evaluated using principal-component analysis (embedded in the DESeq2 package), and genes were deemed to be significantly expressed if the log_2_ fold change was greater than or equal to 1 or less than or equal to −1 and with an adjusted *P* value (*P*_adj_) value of <0.01. Gene set enrichment analysis (GSEA) (57) and Ingenuity pathway analysis (Qiagen) (58) software were used to compare gene sets that were differentially regulated after infection with WT and Δ*GRA28* parasites.

### CCL22 and Ccl22 ELISA.

CCL22/Ccl22 ELISAs were performed on culture supernatants (undiluted or diluted when necessary) using Immulon 4HBX flat-bottom microtiter plates with the human CCL22/MDC DuoSet ELISA (R&D Systems DY336) or the mouse Ccl22/Mdc DuoSet ELISA (R&D Systems DY439) per the manufacturer’s instructions.

### Mouse experiments with WT and Δ*GRA28* parasites.

To determine the impact of T. gondii candidate effectors on mouse morbidity and cytokine production, BALB/cJ mice from Jackson Laboratories (4 to 6 weeks old, female) were injected intraperitoneally with 200 μl of PBS containing 1.0 × 10^6^ T. gondii tachyzoites or with 200 μl of 0.22-μm-pore-size-filtered parasite solution as a mock treatment. Mice were sacrificed at 48 h postinfection (hpi), and PECs were collected by injecting 3 ml sterile PBS into the abdominal cavity, rocking the mouse to mix the PBS, and siphoning the PBS solution into a sterile conical tube. The solution was then centrifuged at 1,000 × *g* for 10 min at 24°C, the supernatant was collected, and RNA was extracted from the pellet for reverse transcription-quantitative PCR (RT-qPCR) and RNA-seq. Blood was collected in Sarstedt Microvette CB 300 Z tubes by cardiac puncture and centrifuged at 10,000 × *g* for 5 min to separate the serum. Mice were infected with RHΔ*HPT* (wild type), RHΔ*MYR1*, or RHΔ*GRA28* parasites depending on the experiment.

To determine the impact of *GRA28* deletion on T. gondii proliferation and dissemination, female BALB/cJ mice (4 weeks old) from Jackson Laboratories were injected intraperitoneally with 200 μl PBS containing 100 T. gondii tachyzoites. In one experiment, five mice received an injection of RHΔ*GRA28* parasites, while the other five received an injection of the transfection control parasite that was transfected with empty vector (RHΔ*HPT:HPT*). For behavioral indices of inflammatory responses, photographs of the mice were taken dorsally and laterally every 4 to 6 h for the entire duration of the infection. Mice were visually scored 0 to 3 based on the presence of fur ruffling, the location of ruffling, and the presence of skin redness/irritation (Fig. S8), as follows: 0, no fur ruffling or red/irritated skin present; 1, mild ruffling present, located predominantly on the head and back of the neck; no red/irritated skin visible; 2, moderate ruffling present—fur forms larger clumps and extends to the rest of the body; skin may be visible through the clumps but is not red or irritated; 3, severe ruffling characterized by fur ruffling present across the entire body with visibly red/irritated skin in between fur clumps.

In a second experiment, mice were infected with 1,000 tachyzoites of T. gondii strain ME49:*LUC*Δ*GRA28* or a passage-matched wild-type strain (27, 28). Mice were imaged daily after injection of d-luciferin as described previously (28, 59) using an IVIS Lumina II *in vivo* bioluminescence imaging system (with ventral imaging occurring on all days postinfection and ventral and dorsal imaging occurring starting on day 10 postinfection). Animals were anesthetized using 2% isoflurane during the 4- to 8-min imaging period (ventrally and dorsally where applicable). When necessary, blood was collected via submandibular lancet puncture, collected into Sarstedt Microvette CB 300 Z tubes, and spun at 10,000 × *g* for 5 min to separate the serum. Mice were monitored extensively over the course of infection for symptoms of morbidity and humanely euthanized. All animal procedures were approved by the Division of Laboratory Animal Resources and IACUC, and our animal facilities are routinely inspected by the USDA and the local IACUC.

### Statistics.

All statistics were performed in GraphPad Prism for Windows (versions 7 or 9; GraphPad Software, La Jolla, CA). For most two-treatment assays, we used an unpaired, two-tailed Student's *t* test, and for multitreatment/condition experiments, we used one- or two-way analysis of variance (ANOVA) followed by multiple-comparison *post hoc* tests. Individual comparisons are listed for each assay in the text and figure legend, and only preplanned comparisons were performed to minimize type 1 errors. *In vivo* bioluminescence data (total flux; photons/s) and nuclear staining intensity data (comparing nuclear trafficking of T. gondii GRA28 when expressed in T. gondii or N. caninum) were log_10_ transformed prior to statistical analysis.
