# Supplementary figures and images for: Toxoplasma gondii GRA28 Is Required for Placenta-Specific Induction of the Regulatory Chemokine CCL22 in Human and Mouse
Source: mBio. 2021 Nov 16;12(6):e01591-21. doi: 10.1128/mBio.01591-21 (PMC8593671; doi:10.1128/mBio.01591-21)

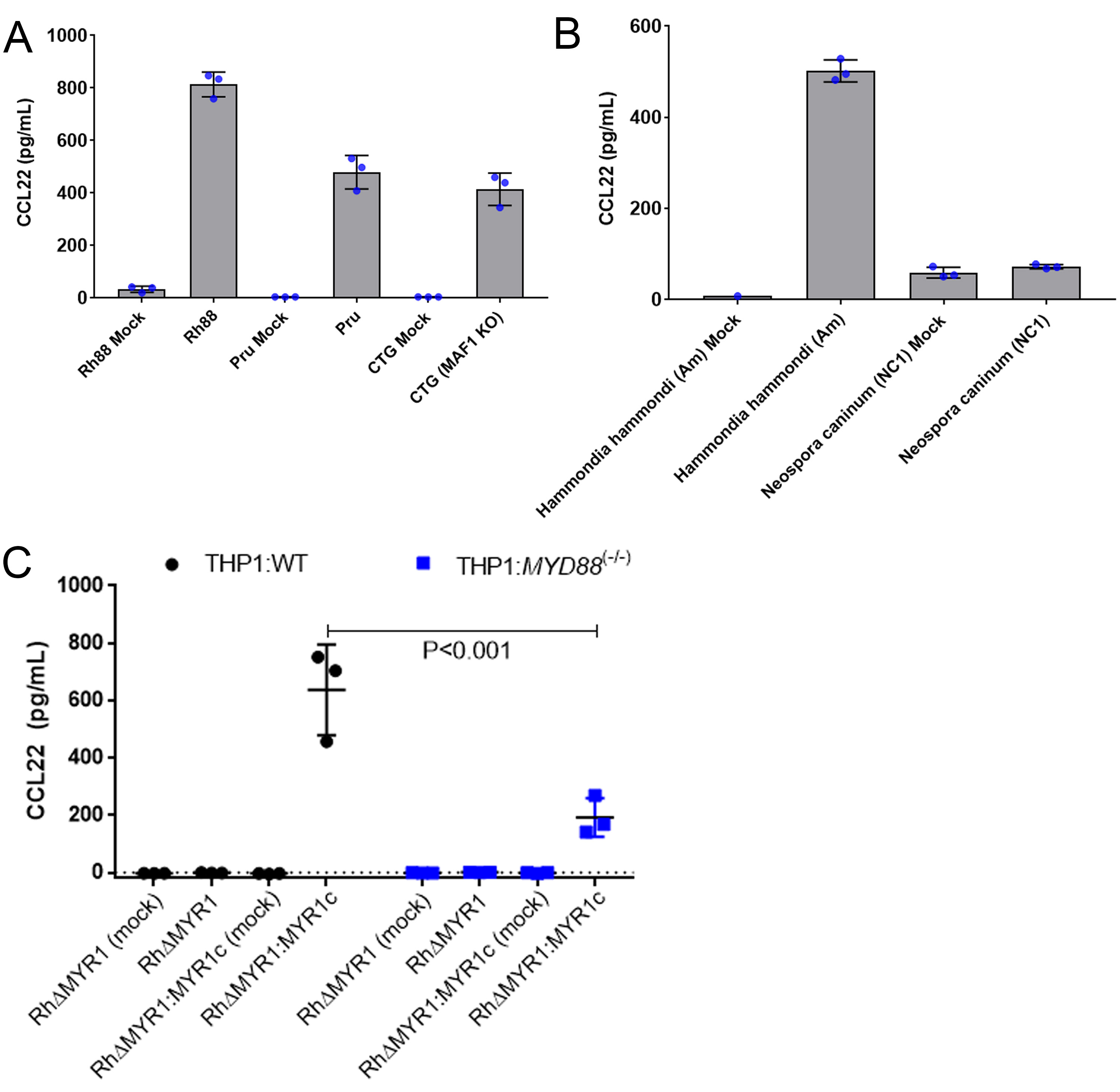

Supplement: FIG S1 [file mbio.01591-21-sf001.jpg]

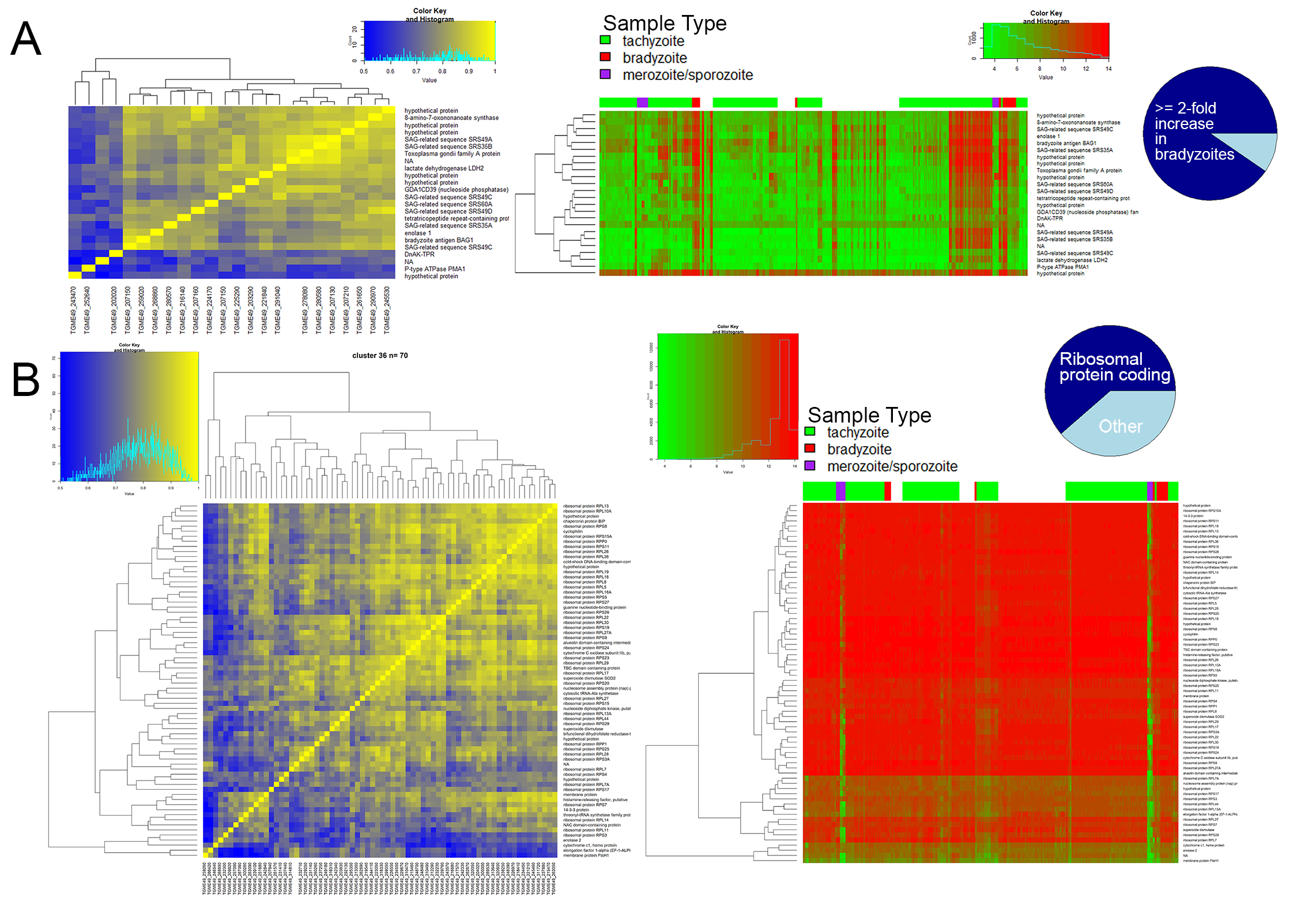

Supplement: FIG S2 [file mbio.01591-21-sf002.jpg]

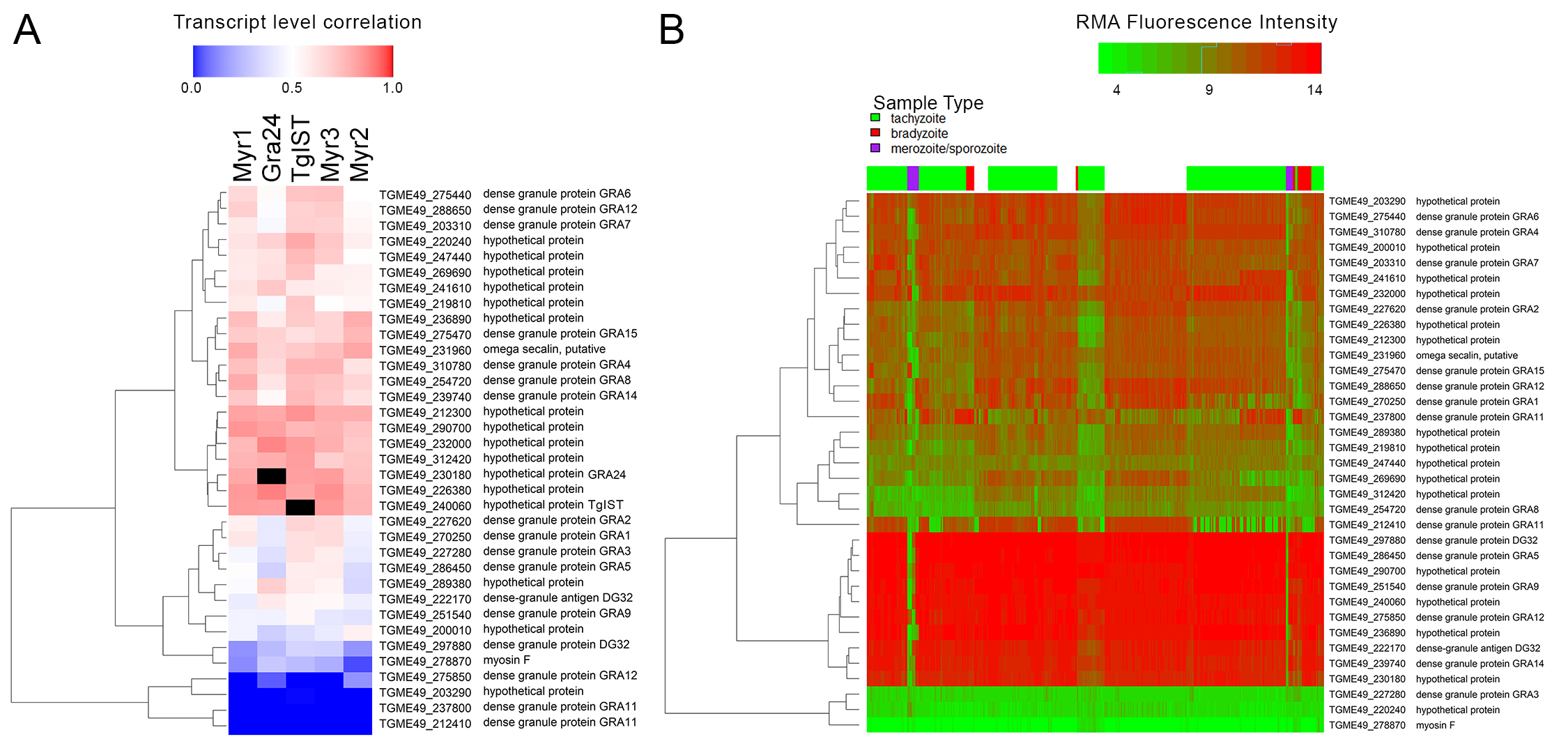

Supplement: FIG S3 [file mbio.01591-21-sf003.jpg]

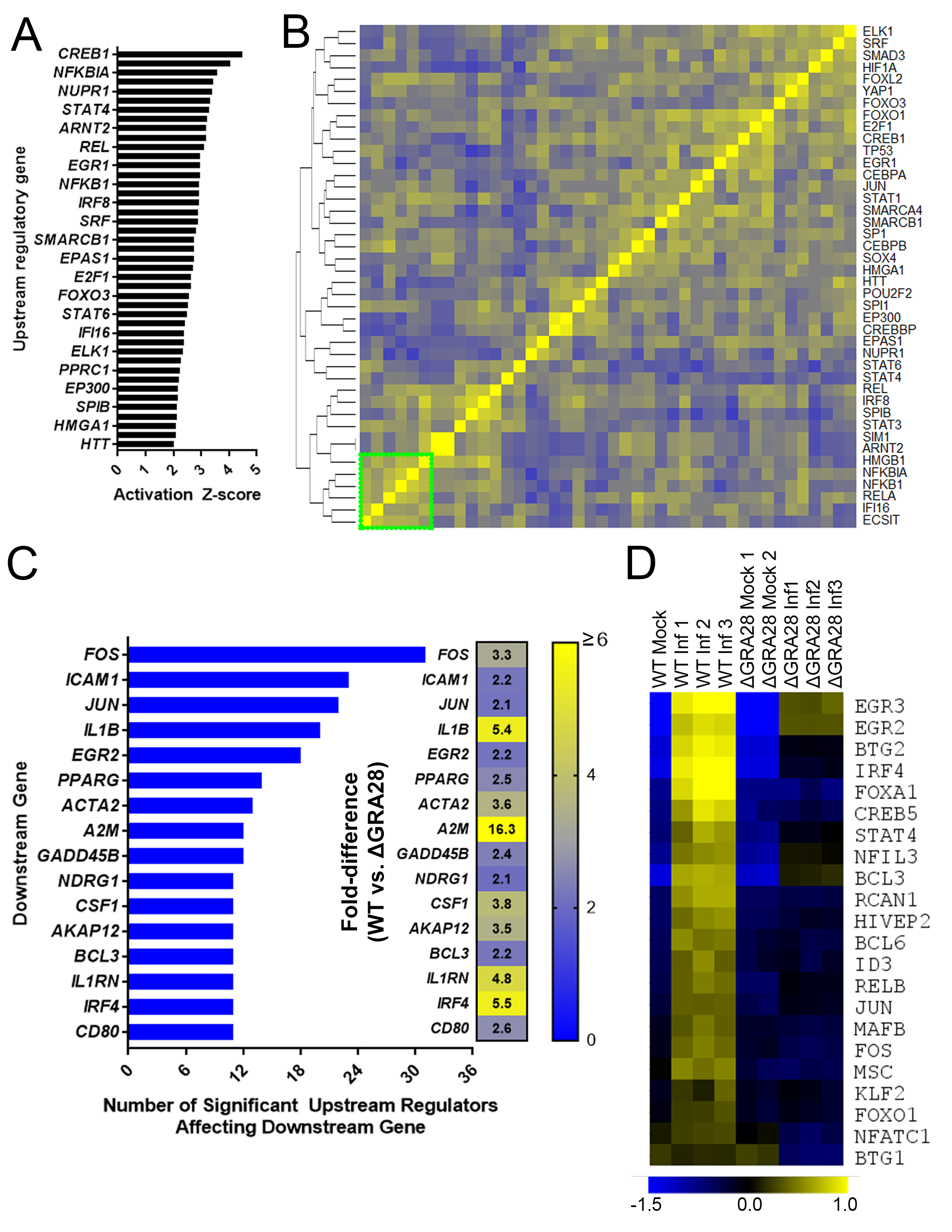

Supplement: FIG S5 [file mbio.01591-21-sf005.jpg]

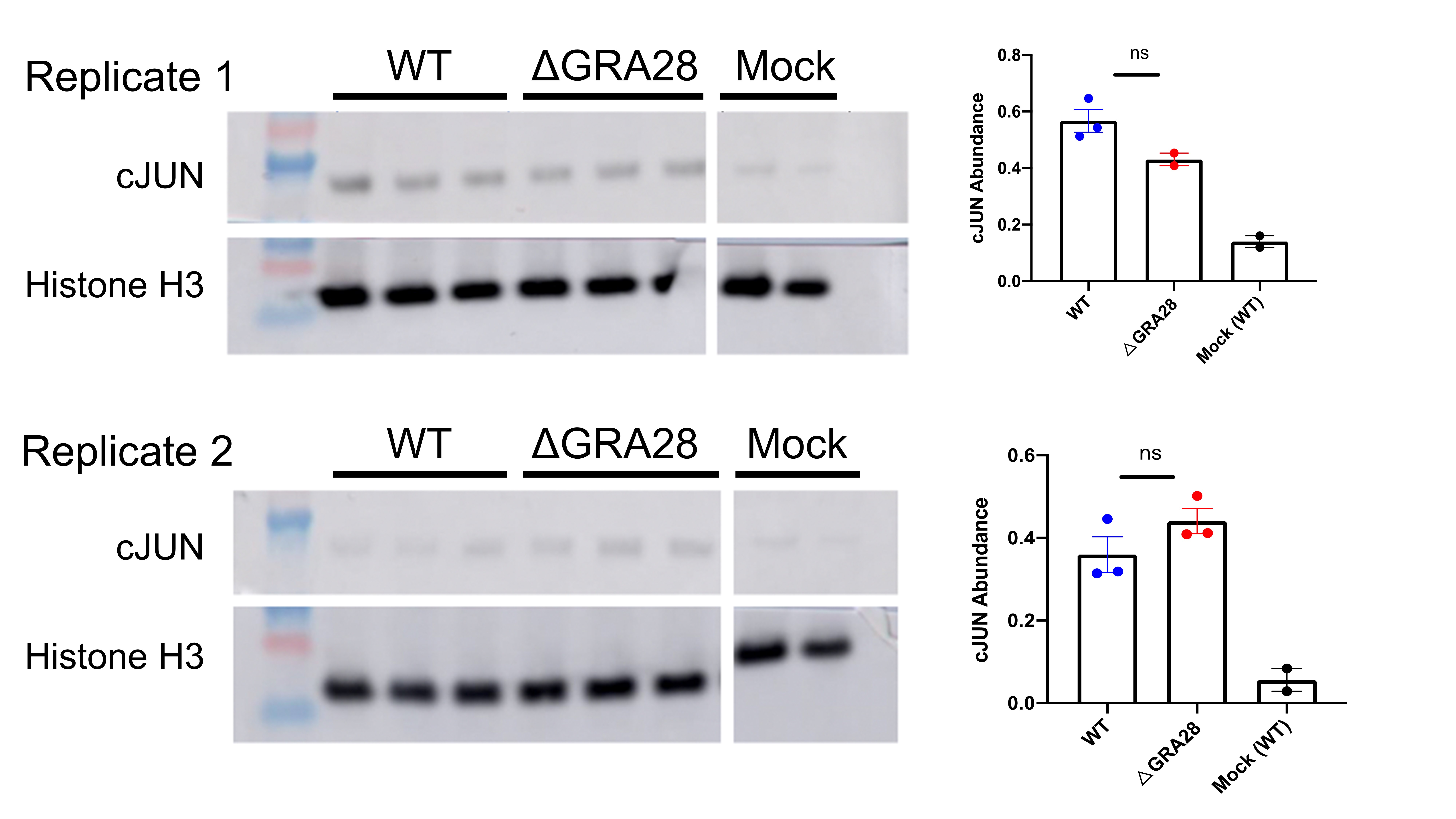

Supplement: FIG S6 [file mbio.01591-21-sf006.jpg]

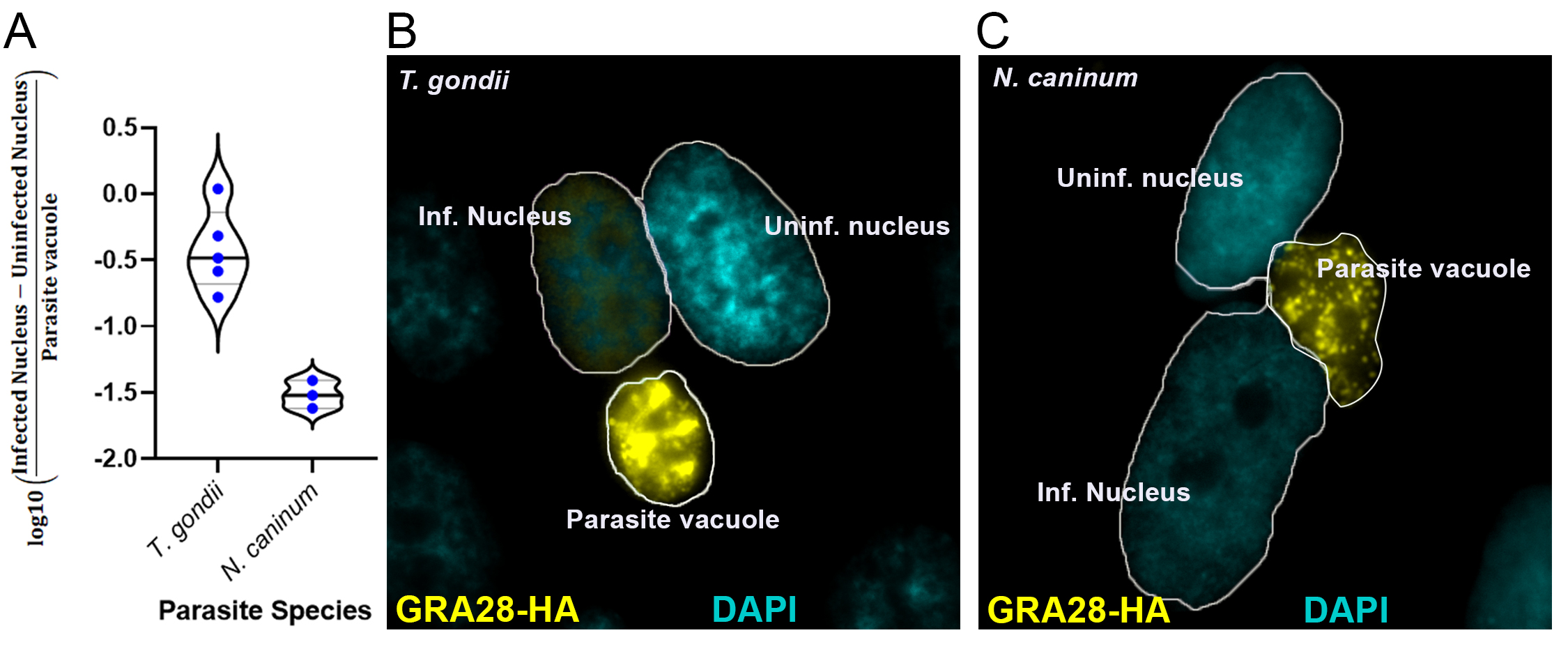

Supplement: FIG S7 [file mbio.01591-21-sf007.jpg]

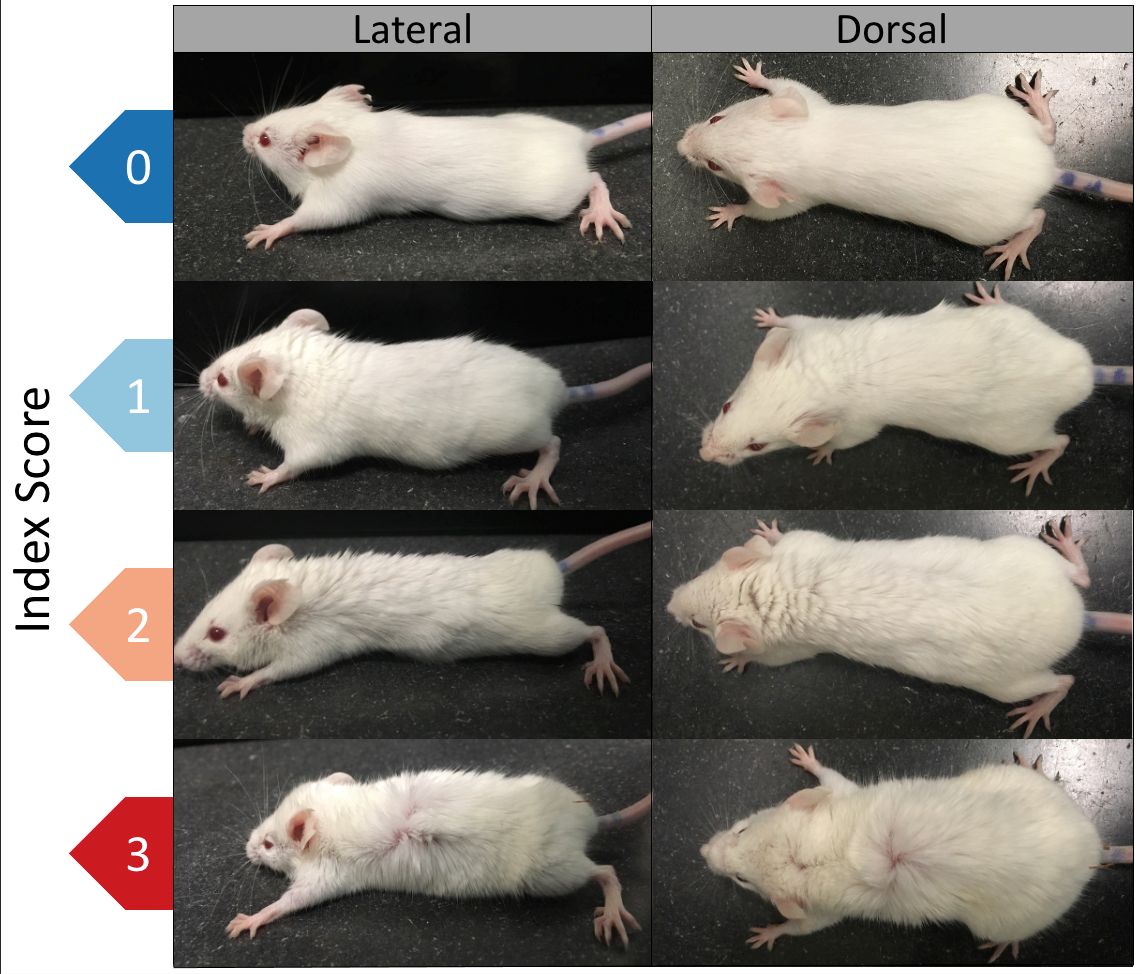

Supplement: FIG S8 [file mbio.01591-21-sf008.tif]
