# Supplementary material for: Toxoplasma gondii GRA28 Is Required for Placenta-Specific Induction of the Regulatory Chemokine CCL22 in Human and Mouse
Source: mBio. 2021 Nov 16;12(6):e01591-21. doi: 10.1128/mBio.01591-21 (PMC8593671; doi:10.1128/mBio.01591-21)
Supplement: FIG S4 [file mbio.01591-21-sf004.pdf]

A

TGGT1_201390 knockout validation
Co-transfected with gRNAs 2 and 12
Clones assayed for figure 3 in ***bold italic***

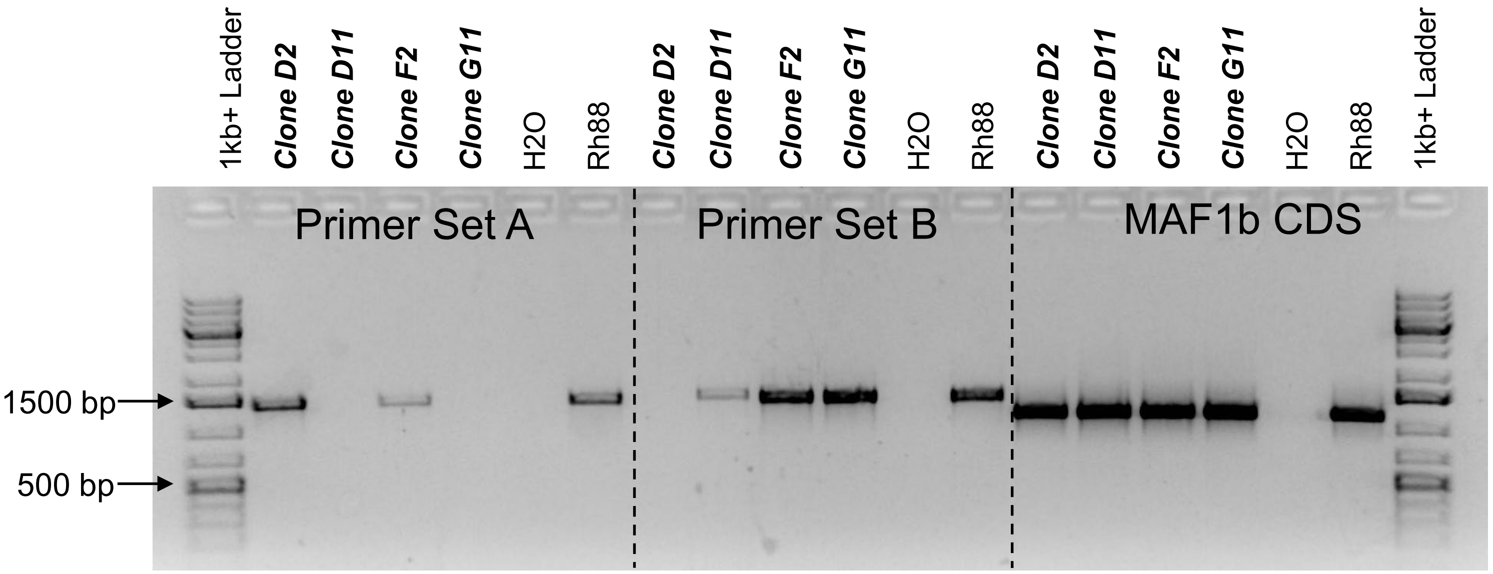

Primer set A: gRNA target site 2 (1406 bp)
Primer set B: gRNA target site 12 (1436 bp)

MAF1b CDS: 1305 bp

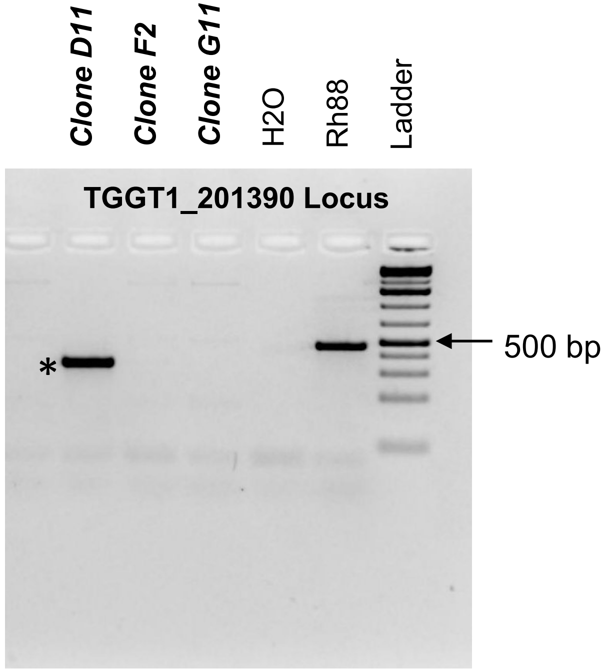

TGGT1_201390
Locus (465 bp)
*Smaller than WT size

B

GRA4 Knockout validation

Co-transfected with guides 0 and 26

Clones assayed for figure 3 in ***bold italic***

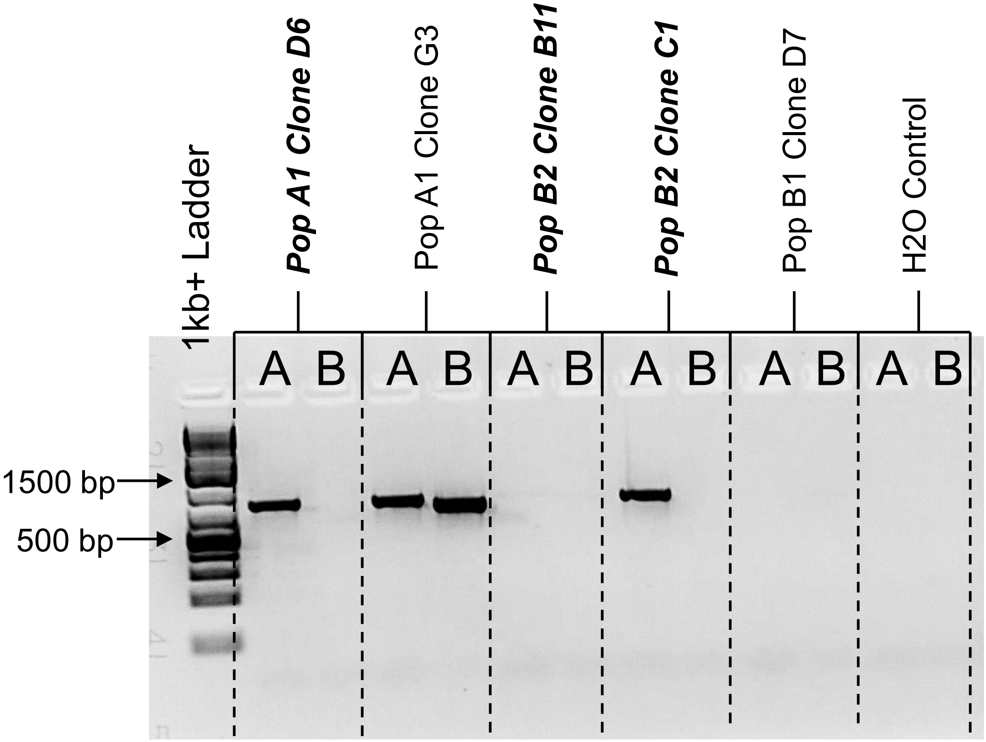

Primer set A: gRNA target site 26 (788 bp)

Primer set B: gRNA target site 0 (738 bp)

C

GRA8 Knockout validation

Transfected with gRNA 4

Clones assayed for figure 3 in ***bold italic***

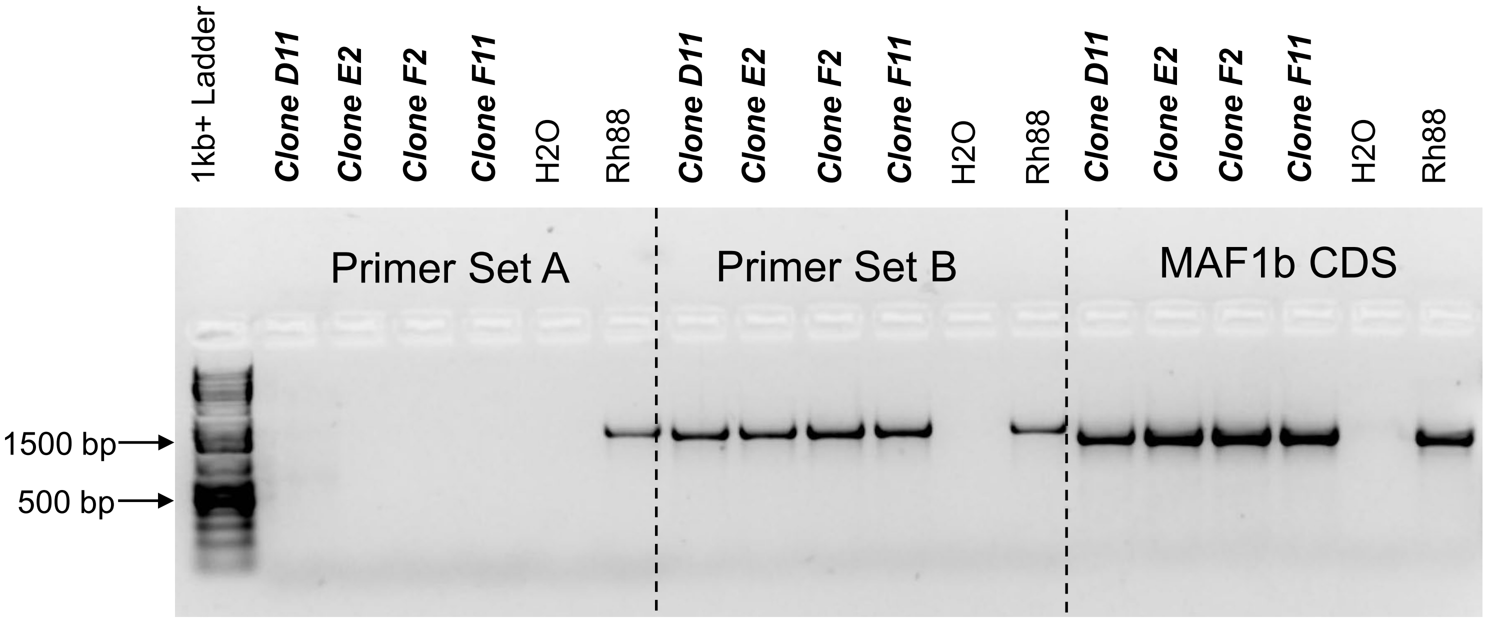

Primer set A: gRNA target site 4 (1448 bp)

Primer set B: gRNA target site 27 (1381 bp)

MAF1b CDS (1305 bp)

D

TGGT1_231960 (GRA28) Knockout Validation
Co-transfected with gRNAs 5 and 26
Clones assayed for figure 3 in ***bold italic****

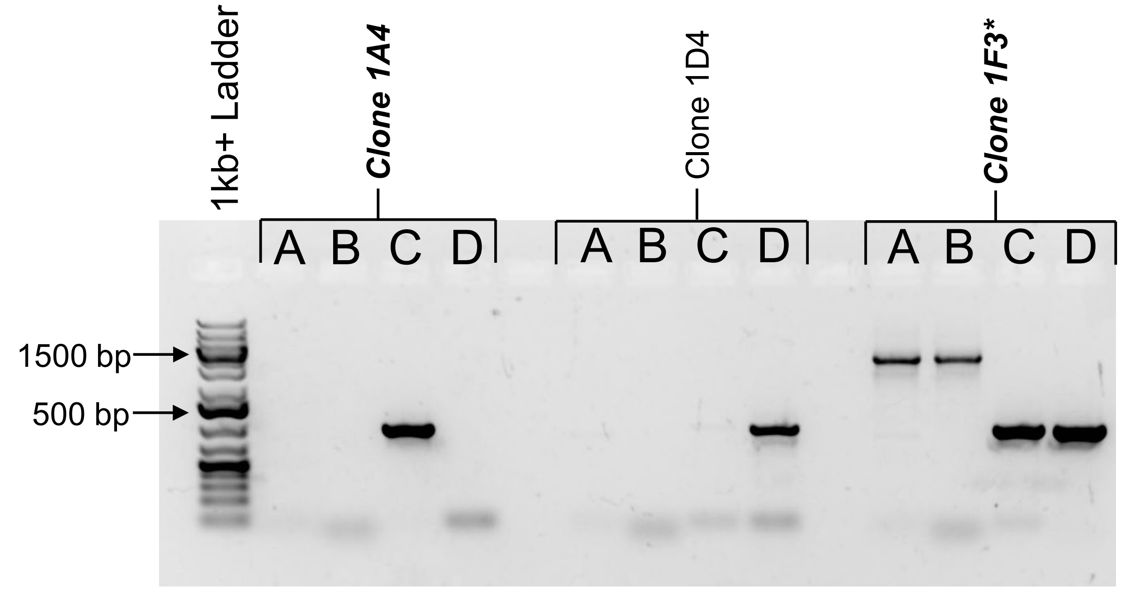

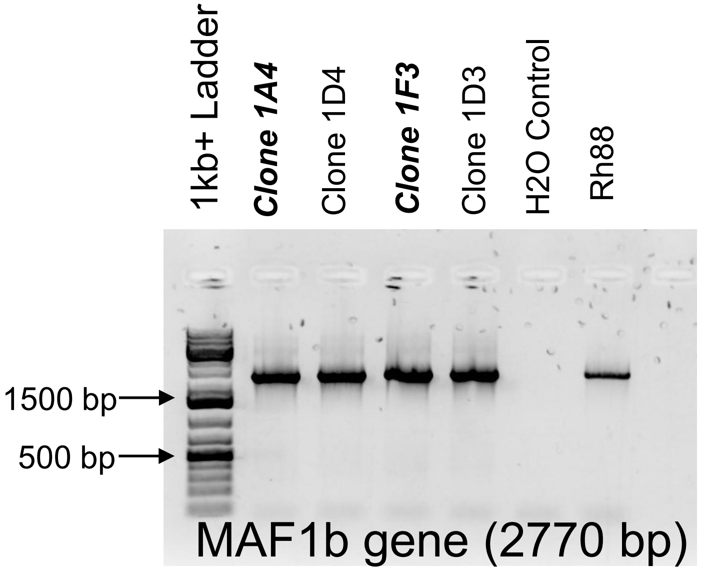

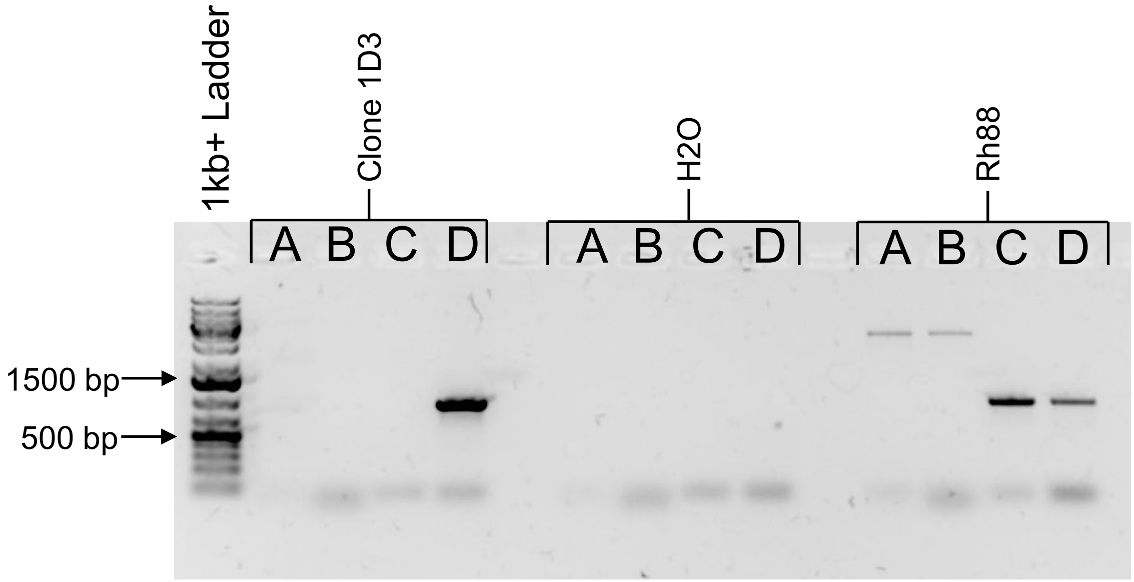

Primer set A: GRA28 gene (4326 bp)
Primer set B: GRA28 gene (4353 bp)

Primer set C: gRNA target site 5 (974 bp)
Primer set D: gRNA target site 26 (966 bp)

*Clone 1F3 has a single deletion in gRNA target site 5 near the PAM (the bottom strand is shown):

gRNA and PAM 5 WT: GTTCCGCTGGTGCCTT**C**ACC [TGG]
gRNA and PAM 5 1F3: GTTCCGCTGGTGCCTT-ACC [TGG]

This results in a premature stop codon 100 bp downstream of the CRISPR-driven deletion.

E GRA18 Knockout Validation

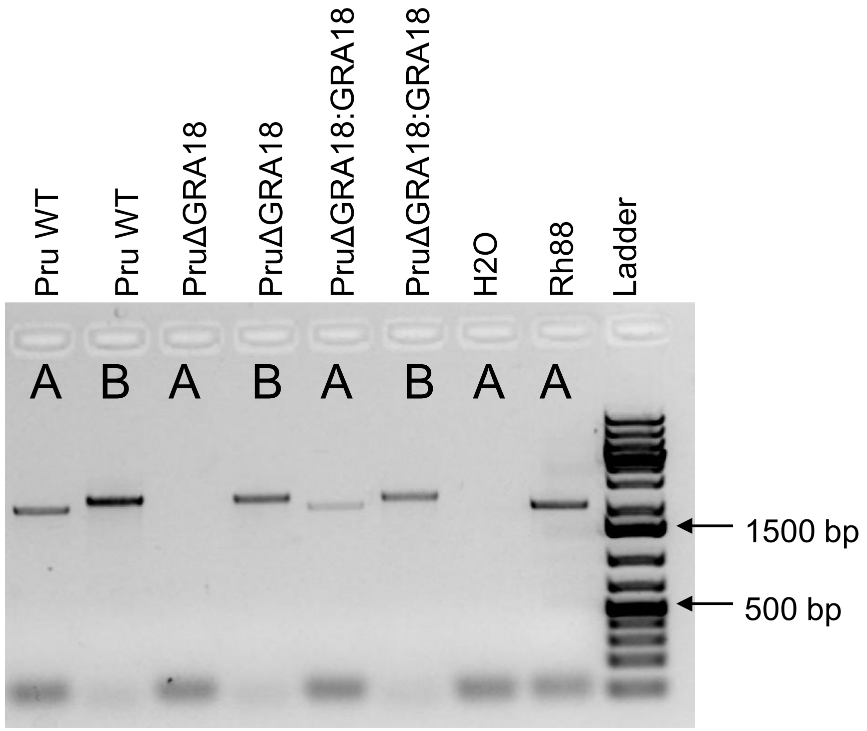

Primer set A: GRA18 gene (2137 bp)
Primer set B: MAF1b gene (2770 bp)

**Strains same as those used in He et al., eLife 2018.*
