# Supplementary material for: Toxoplasma gondii GRA28 Is Required for Placenta-Specific Induction of the Regulatory Chemokine CCL22 in Human and Mouse
Source: mBio. 2021 Nov 16;12(6):e01591-21. doi: 10.1128/mBio.01591-21 (PMC8593671; doi:10.1128/mBio.01591-21)
Supplement: TABLE S1 [file mbio.01591-21-st001.docx]

| Targeted locus | Locus Primers | gRNA(s) [PAM]* | Primers flanking PAM(s) (to detect and/or sequence insertion) |
| --- | --- | --- | --- |
| TGGT1_201390 | F: GGCGAAGCGTGAGGCAAGCT  R: GGGTGCAAGAGGTAGCCGTG | 2: GGCCAGGCCACAAAGCGCGA [TTG] | F: TTTGCCAAGCCGGAAACTA  R: GTCTTCAACACCGGAGAGTATG |
|  |  | 12: GCAAGGTTAGGCTTCTGGCC [TGG] | F: AGGGTACTCAAGAAGGACTGA  R: AAGACGCAGGTGCAAGAG |
| TgGRA4 | F: CGTTCGAATCGCATGCAACG  R: GGAACATGTAGCGTCCACTG | 0: GTGTCGCATCTTGCGTGTGG [AGG] | F: GCCGGGTGATAATAGGTGGC  R: AACATCTGTACCTGCTTGCG |
|  |  | 26: GTCAAGAAGGGAATCCTGAC [GGG] | F: TGCAACTTGCGGACCTGAAT  R: GTGACCAGGTGTTCCTGGAA |
| TgGRA8 | F: GCAGTTGCATACGCTCATGG  R: GTTTCCGTTCCGGTCACAGG | 4: GGATGGAAACCGGGAGGAGG [AAG] | F: GTGCCACGACTCCTGAAATAG  R: TACTGGAGTACCCACTGGATATG |
|  |  | 27: GCACGGGAGCCACTTCGGGA [CGG] | F: TCCAGTGGGTACTCCAGTAAT  R: CCTGACCTCAGCATGGATTT |
| TgGRA28 | F: CCTGTTGAGTACGCTGTCTT  R: CTCCCTGGTTTGGGACTATTT | 5: GTTCCGCTGGTGCCTTCACC [TGG] | F: GTTCCCGACTGCGAAATAGAA  R: CTGTAACTCACTCTCGCCATTT |
|  | F: CTTCGAAAGGAAATTCGGGAAAC  R: GTGAACCAAGAAGAGGAACATAGA | 26: GTAGACGAACTGCTGCCTTG [GGG] | F: CACCACCGATTGTCTCATAGTC  R: TACCACCTGGGAACGTAAGA |
| TgGRA18 | F: CGAGCCCAGACAGGCGCCCC  R: CGGTGGACTGCATAACATCC | NA** | NA** |

**Table S1: Primer sequences and gRNA protospacer sequences used in the current study**

********gRNA sequence was incorporated into the pSAG:CAS9-GFP plasmid using Q5 mutagenesis and described in methods*

*********homologous recombination was used to delete GRA18; He et al., eLife 2018;7:e39887*
